# Precision engineering of anti-atherosclerotic herbal nanomedicine: from machine learning-aided active components screening to optimized metal-phenolic network codelivery

**DOI:** 10.1007/s13346-025-02023-3

**Published:** 2025-12-04

**Authors:** Yao Chen, Meiting Lu, Lu Zhang, Errong Chang, Qinglan Zhu, Qianlan Xu, Ziting Gao, Dongmei Pan, Chunyan Shen, Qiang Liu, Zhong Zuo, Cuiping Jiang

**Affiliations:** 1 Guangdong Provincial Key Laboratory of Chinese Medicine Pharmaceutics, School of Traditional Chinese Medicine, Southern Medical University, Guangzhou, 510515 People’s Republic of China; 2 School of Pharmacy, Faculty of Medicine, The Chinese University of Hong Kong, Hong Kong SAR, People’s Republic of China; 3 Laboratory Animal Center, Southern Medical University, Guangzhou, 510515 People’s Republic of China

**Keywords:** Metal-phenolic network, Polyphenols, Codelivery system, Machine learning, Atherosclerosis

## Abstract

Atherosclerosis (AS), a chronic inflammatory disease linked to oxidative stress and lipid imbalance, remains a major cardiovascular threat. Traditional herbs *Salvia miltiorrhiza* and *Carthamus tinctorius* exhibit multi-target anti-AS potential, yet their compositional complexity limits clinical translation. This study aimed to systematically identify core anti-AS components from these herbs and enhance their anti-AS efficacy via machine learning-aided screening and nanotechnology-driven codelivery. We initially pioneered a machine learning-aided hybrid strategy integrating network pharmacology and quantitative activity relationship (QSAR) modeling to identify four core anti-AS polyphenols (i.e., salvianic acid A, salvianolic acid B, protocatechuic acid, and hydroxysafflor yellow A). Subsequently, a quaternary metal-phenolic network (SSPH-MPN) was engineered for plaque-targeted codelivery, optimized via the median-effect principle for achieving a synergistic effect based on ROS scavenging efficacy. The optimized SSPH-MPN was characterized by a series of studies, including molecular dynamics simulations, UV, DLS, TEM, FTIR, XPS, and ICP-MS. The anti-AS effect of the optimized SSPH-MPN was evaluated by monitoring oxidative status (ROS levels, antioxidant enzymes SOD, GSH-Px, MDA, T-AOC), inflammatory markers (IL-1β, IL-6, TNF-α), lipid metabolism (DiI-oxLDL uptake, cholesterol efflux, blood lipid levels, lipid accumulation), and plaque areas. The results demonstrated that the optimized SSPH-MPN showed great efficiency in inhibiting lipid uptake and accumulation, and mediating cholesterol efflux in RAW 264.7 cells, and exhibited improved lipid metabolism, attenuated oxidative stress and inflammation, thus acquired diminished plaque area in apoE^-/-^ mice. Furthermore, biocompatibility was assessed through hemolysis, cytotoxicity assays, and in vivo safety studies, confirming its suitability as a safe therapeutic agent. In conclusion, this work not only identified four anti-AS polyphenols from traditional herbs but also established an MPN-based co-delivery system for synergistic anti-AS therapy, providing a comprehensive paradigm from drug discovery to formulation development.

**Graphical Abstract:**

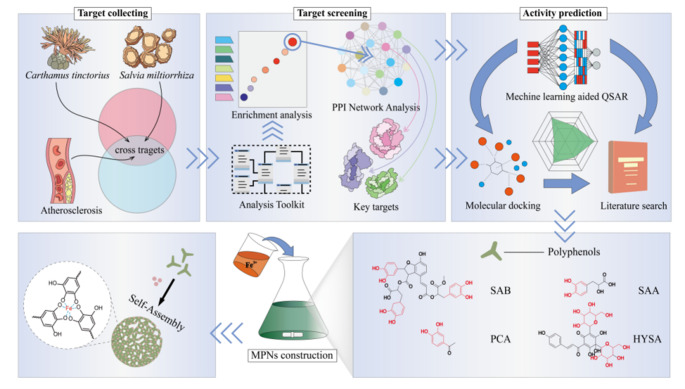

**Supplementary Information:**

The online version contains supplementary material available at 10.1007/s13346-025-02023-3.

## Introduction

Atherosclerosis (AS) is a chronic inflammatory vascular disease characterized by intimal thickening and lumen narrowing within the arteries. This pathological condition is the underlying cause for cardiovascular complications, such as stroke, ischemic heart disease, acute myocardial infarction, etc [1, 2]. The pathogenesis of AS is multifaceted, primarily associated with inflammation, oxidative stress, and lipid infiltration, resulting in endothelial dysfunction, monocyte adhesion, formation of lipid-laden macrophage foam cells, as well as plaque formation or even rupture. Consequently, there is clearly an urgent need for a drug combination with multiple pharmacological activities against AS so as to achieve synergistic anti-AS efficacy [3].

The use of traditional Chinese medicine (TCM) in AS prevention and treatment has gained widespread recognition. Several herbal formulas containing the herb pair of *Salvia miltiorrhiza and Carthamus tinctorius* have proven to be notably effective in the treatment of AS. For instance, Guanxin II Decoction and Danhong Injection have a long-standing history in clinical practice, demonstrating favorable outcomes in managing AS [4, 5]. Such success is primarily due to the multiple ingredients from *Salvia miltiorrhiza* and *Carthamus tinctorius*, which possess synergistic anti-atherosclerotic properties, including antioxidative, anti-inflammatory, and lipid-regulating activities. Since the compositional complexity of TCM formula containing *Salvia miltiorrhiza* and *Carthamus tinctorius* limits their global usage due to the difficulties in quality control, screening of their core active components with synergistic anti-AS effect serves as a critical prerequisite for further development of drug formulation for AS treatment.

To identify potential active components in TCM, network pharmacology-based screening became an emerging tool due to its capabilities of analyzing biomolecular networks, uncovering the multi-level interactions between drugs and biological systems [6, 7]. Such a tool starts with identifying cross target genes between TCM and the disease, leveraging big data to select key therapeutic pathways, such as enrichment analysis and protein-protein interaction (PPI) networks, to pinpoint core therapeutic targets. Subsequently, these identified targets are used to determine the active components through molecular docking. However, molecular docking typically yields only qualitative interaction data, which is often insufficient for the optimization of the screening process [8, 9]. Therefore, establishing quantitative activity relationships (QSAR) between components and core targets is necessary to enhance the precision of the analysis. QSAR is a mathematical model linking chemical structures (via molecular descriptors) to biological activities or physicochemical properties, addressing the lack of quantitative analysis in network pharmacology [9, 10]. Recently, machine learning algorithms such as support vector machine, random forest, and decision tree are increasingly used to build non-linear QSAR model to enhance its predictability [11, 12]. Consequently, integrating network pharmacology with machine learning-enhanced QSAR can enable rapid and accurate identification of active components in TCM.

A challenging issue to translate the identified active components from TCM into related therapy is to ensure their sufficient in vivo exposures. Due to the well-known limited bioavailability of active components in TCM [13], nanotechnology-based delivery systems have emerged as a promising solution, offering enhanced stability, prolonged half-life, and targeted drug delivery [14]. Among these delivery systems, the metal-phenolic network (MPN) utilizes the ortho-dihydroxy groups in active components to coordinate with metal ions [15], forming nanoparticles that effectively address the low bioavailability of compounds like polyphenols, flavonoids, and terpenoids. Chen et al. leveraged this principle to enhance the bioavailability of Curcumin, remarkably improving its therapeutic efficacy in cancer treatment [16]. Furthermore, several studies have employed PEG-polyphenols to fabricate MPN for surface modification, effectively increasing the targeting capabilities [17, 18]. These findings highlight MPN-based nanotechnology is a promising approach to enhance the bioavailability of active components, offering a versatile platform for targeted drug delivery [19].

**Scheme 1:**
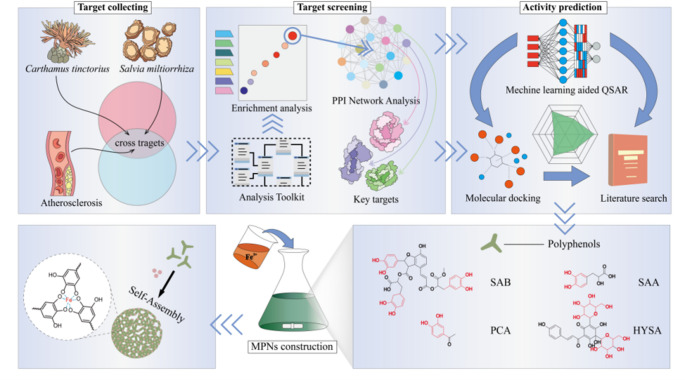
The schematic illustrates the process of screening core components from *Salvia miltiorrhiza* and *Carthamus tinctorius* and their assembly into MPN nanoparticles

Our current study aimed to (i) systematically identify core anti-AS components from *Salvia miltiorrhiza* and *Carthamus tinctorius* via machine learning-aided screening and (ii) enhance their anti-AS efficacy via MPN-based codelivery system, as highlighted in Scheme 1. To achieve these objectives, a machine learning-aided hybrid method integrating network pharmacology and QSAR modeling was established to screen the active components of *Salvia miltiorrhiza* and *Carthamus tinctorius*. Besides, the identified key components were assembled into a quaternary MPN through coordination with metal ions, with the formulation optimized via the median-effect principle. The optimized MPN was characterized, followed by evaluations of their plaque targeting efficiency, antioxidative activities and biocompatibility. Furthermore, in vitro and in vivo anti-AS effects were evaluated via Oil Red O staining, cholesterol removal, and DiI-oxLDL uptake assays, and monitoring of serum inflammatory cytokine, oxidative status, and lipid levels.

## Materials and methods

### Materials

Hydroxysafflor yellow A (HSYA, 98%) and salvianolic acid B (SAB, 98%) were obtained from Alfa Bio-Technology Co., Ltd (Chengdu, China). Protocatechuic aldehyde (PCA, ≥ 98%), salvianic acid A (SAA, ≥ 98%), and rhodamine 123 (R123) were bought from Yuanye Bio-Technology Co., Ltd (Shanghai, China). LysoTracker Red DND-99 was obtained from Yeasen Bio-Technology Co., Ltd (Shanghai, China). An active oxygen detection kit and DAPI were bought from Biyuntian Biotech Co., Ltd. (Shanghai, China). 3-(4, 5-Dimethylthiazol-2-yl)-2,5-diphenyltetrazolium bromide (MTT), oil Red O and FeCl_3_.6H_2_O were obtained from Aladdin Chemical Reagent Co., Ltd (Shanghai, China). BODIPY-cholesterol was purchased from Bidepharm Co., Ltd (Shanghai, China). PVP K-30 was obtained from MYM biological technology company Co., Ltd (Shanghai, China).

### In silico screening of active components from *Salvia miltiorrhiza* and *Carthamus tinctorius*

#### Collection and screening the cross-targets between active components and AS

The active components from *Salvia miltiorrhiza* and *Carthamus tinctorius* were retrieved from the Chinese Medicine System Pharmacological Database and Analysis Platform (TCMSP, http://lsp.nwu.edu.cn/tcmsp.php) based on the criteria of oral bioavailability (OB) ≥ 30% and drug-likeness (DL) ≥ 0.8, and their associated target genes were then collected. These target genes were normalized and imported into the UniProt database (https://www.uniprot.org/) to obtain UniProt IDs. After consolidating the UniProt IDs, duplicates were removed to construct the component gene dataset for the two herbs. To identify AS-related genes, the keywords “atherosclerosis” and “atherosclerotic plaque” were used to query four databases: GeneCards (https://www.genecards.org/), Online Mendelian Inheritance in Man (OMIM, https://omim.org/), Therapeutic Target Database (TTD), and Pharmacogenetics and Pharmacogenomics Knowledge Base (PharmGKB, https://www.pharmgkb.org/). The target genes from these four databases were normalized and imported into the UniProt database to retrieve UniProt IDs, which were consolidated and filtered to remove duplicates, to acquire the AS-related gene dataset. Finally, a Venn diagram was used to identify the intersection between the component gene dataset and the AS-related gene dataset.

#### KEGG enrichment analysis and PPI network visualization for the cross targets

The Kyoto Encyclopedia of Genes and Genomes (KEGG, https://www.genome.jp/kegg/) is a database used for systematic gene function analysis and annotation. KEGG enrichment analysis was conducted using the BiocManager package in R to further analyze the intersection between the component gene dataset and the AS-related gene dataset. The resulting gene pathways were ranked based on their low p-values, with key pathways related to the treatment of AS being identified. Among the top-ranked pathways, 35 target genes were found to be associated with this condition. PPI data for these targets were retrieved using STRING (https://string-db.org/), with the organism specified as Homo sapiens and the interaction score filtered for high confidence (≥ 0.900). Cytoscape 3.7 was employed to construct and visualize the PPI network.

#### Therapeutic target activity data collection for the QSAR model construction

The 35 genes were queried in the UniProt database (https://www.uniprot.org/) to obtain corresponding target names. These target names were subsequently input into the ChEMBL database (https://www.ebi.ac.uk/chembl/), with the search restricted to single protein targets and the organism limited to Homo sapiens. Experimental data on active molecules associated with these 35 therapeutic targets were retrieved, including IC50, activity levels, and inhibition rates. Compounds with molecular weights ranging from 0 to 500 nM were selected to ensure clinical relevance. Duplicate records were removed to maintain data independence and accuracy. For datasets with fewer than 20 activity data points, model construction and prediction were excluded due to limited training data. Ultimately, twelve datasets were chosen for the construction of machine learning models.

#### Chemical descriptor calculation

The retrieved activity data were matched with the corresponding SMILES and converted into “.smi” files using RDKit (https://www.rdkit.org/). Chemical descriptors for each active compound were then calculated using the Mordred descriptor calculator (https://github.com/mordred-descriptor/mordred-web) to establish a descriptor-activity dataset for each target.

#### Establishment of machine learning-based QSAR models

Machine learning models were then constructed to predict pharmacological activity built on the QSAR datasets. Briefly, for each target, after calculating the chemical descriptors of the collected molecular activity data using the previously described method, these descriptors served as features, while the activity values were used as labels for training. To ensure the comparability of activity values across different targets, the data were processed according to the target’s effect on AS improvement. The activity data were processed in various ways to obtain a standardized score on a 10-point scale. The data were cleaned and partitioned into training and test sets at a 4:1 ratio. Cross-validation was conducted on the training set using various algorithms, including LsBoost, Gaussian regression, random forest, decision tree, support vector machine (SVM), and partial least squares (PLS), to optimize the models. LsBoost was configured with default parameters, Gaussian regression with a noise standard deviation (σ) of 0.1, random forest with a maximum tree depth of 500 and a minimum leaf node count of 5, and a decision tree with 10 leaf nodes. Default parameters were used for SVM and PLS. Based on these models, the pharmacological activity of active compounds in TCM against the targets was predicted, and the output values were prepared for subsequent pharmacological analysis and validation.

#### Hyperparameter optimization of LsBoost

To systematically optimize model performance, we employed a two-stage grid search strategy for hyperparameter tuning. Three critical parameters were selected for optimization: the number of learning cycles (NumCycles), learning rate (LearnRate), and minimum leaf size (MinLeafSize). The optimization began from a baseline configuration using default parameters (NumCycles = 10, LearnRate = 1.0, MinLeafSize = 1).

In Stage 1, we conducted a coarse-grid search across a broad parameter space to identify high-performance regions. The search ranges encompassed NumCycles from 10 to 500, LearnRate from 0.01 to 1.0, and MinLeafSize from 1 to 20, resulting in 294 unique parameter combinations. To maintain computational efficiency during this exploratory phase, model performance was evaluated using the mean R^2^ value from ten independent runs with different random seeds, rather than the standard one thousand runs employed for final model assessment.

Stage 2 implemented a refined grid search within the promising parameter regions identified in Stage 1. The search space was narrowed to NumCycles ranging from 150 to 250 (step size of 10), LearnRate from 0.35 to 0.55 (step size of 0.01), and MinLeafSize from 6 to 12, yielding 3087 parameter combinations. In addition to predictive performance, we incorporated computational efficiency by monitoring training time for each configuration. The optimal parameters were determined to be NumCycles = 200, LearnRate = 0.50, and MinLeafSize = 10, achieving an R^2^ of 0.9822 with a training time of 0.829 s per model. This optimized configuration demonstrated a 49.64% improvement over baseline parameters and represented a 1.21% enhancement beyond the best Stage 1 result. All final model evaluations and predictions were conducted using one thousand independent runs with the optimized parameters to ensure robust performance estimation.

#### Predicting compounds’ activity based on the machine learning-based QSAR models

The LsBoost model demonstrated the best performance among all the models and was thus used to construct the QSAR model for predicting the activity of the components in *Salvia miltiorrhiza* and *Carthamus tinctorius*. Specifically, the components from these plants were collected and categorized into groups such as terpenoids, flavonoids, polyphenols, steroids, essential oils, quinones, and alkaloids to facilitate subsequent analysis. Chemical descriptors for these components were calculated using the method described in the “Chemical descriptor calculation” section, and these descriptors were used as features in the optimized QSAR model based on the LsBoost algorithm for each target. The predicted activity values for each component against each target were then obtained. The average activity prediction value for each component across different targets was calculated, and the components were ranked according to these average values.

#### Molecular docking-assisted validation of the selected active compounds

The top 50 compounds were selected as ligands by ranking the predicted activity values using the LsBoost algorithm based on QSAR. The molecular docking procedure was performed using the Molecular Operating Environment (MOE) software to quantify the binding affinities between the compounds and target proteins. To ensure consistency between the QSAR model and molecular docking targets, all identified target proteins were retrieved from the Protein Data Bank (PDB, https://www.rcsb.org/) using the following criteria: the source organism was specified as Homo sapiens, the experimental method was limited to X-ray diffraction, and the resolution was ≤ 3 Å. The most recently published protein structure meeting these criteria was selected as the receptor for molecular docking, with a detailed correspondence table of target genes and receptors provided in the supplementary materials.

Protein structures were imported into the MOE software, where redundant conformations and water molecules were removed. Binding sites within the protein pockets were predicted, excluding the original ligand. The top 50 compounds were docked to these binding sites using the Triangle Matcher method. The scoring function was set to London dG, and refinement was performed with the Rigid Receptor approach. Binding affinity scores were recorded and analyzed. The absolute value of the binding affinity represents the binding strength between the ligand and receptor. Larger absolute binding affinity values (which are negative) indicate a stronger binding capacity.

#### Selection of active ingredients via a comprehensive scoring system

Based on the QSAR prediction results, the average values for the top 50 components across 12 targets were calculated and then normalized to obtain Score1. Additionally, the absolute values of the binding affinity for 10 targets of these components were averaged and normalized to yield Score2. The final score for each component was calculated as the average of Score1 and Score2 since QSAR predictions and molecular docking represent independent computational methodologies that probe different aspects of compound quality. The top five components with the highest scores were identified as lignan, salvianolic acid B, salvianolic acid E, hydroxysafflor yellow A, and salvianolic acid H. Based on a comprehensive literature review and the key marker compounds of *Salvia miltiorrhiza* and *Carthamus tinctorius* as outlined in the 2025 edition of the Chinese Pharmacopoeia, salvianolic acid B (SAB) and hydroxysafflor yellow A (HSYA) were identified as the most promising candidates for delivery. Furthermore, molecular fragment analysis of the top 50 components revealed that the five most frequently occurring structural fragments were most closely matched by salvianic acid A (SAA) and Protocatechuic aldehyde (PCA), which are key compounds in *Salvia miltiorrhiza* and *Carthamus tinctorius*, respectively.

#### Selection of a combination dose of the active ingredients via calculation of the median-effect doses for the four active components by intracellular ROS scavenging tests

RAW264.7 cells were seeded in 12-well plates (2 × 10^5^ cells/well) and cultured for 12 h. Subsequently, the cells underwent exposure to varying concentrations of SAA (0.6, 2.3, 4.7, 7.0, 11.7 µg/mL), SAB (0.6, 1.1, 2.3, 3.4, 4.6 µg/mL), PCA (0.1, 0.6, 2.3, 3.4, 4.6 µg/mL), and HSYA (0.6, 1.2, 2.4, 3.6, 4.7 µg/mL), respectively. After a 24 h incubation, the medium was then discarded, and the cells were incubated for an additional 12 h in DMEM supplemented with oxLDL (40 µg/mL). Afterwards, the cells were treated with 10 µM of DCFH-DA for 20 min, followed by washing with PBS and collecting by centrifuge three times (2000 × g, 10 min). The mean fluorescence intensity was measured by flow cytometry at the excitation wavelength of 488 nm and emission wavelength of 525 nm. The OxLDL-treated group (without drug intervention) served as the positive control, whereas untreated cells were designated as the negative control. The effect (*fa*) on inhibiting intracellular ROS production was expressed using the following equation:

1 $$\:fa=\frac{{F}_{\mathrm{m}\mathrm{a}\mathrm{x}}-{F}_{\mathrm{s}\mathrm{a}\mathrm{m}\mathrm{p}\mathrm{l}\mathrm{e}}\:}{{F}_{\mathrm{m}\mathrm{a}\mathrm{x}}-{F}_{\mathrm{m}\mathrm{i}\mathrm{n}}}$$

Where F_max_ is the fluorescence intensity of the positive control, F_min_ is the fluorescence intensity of the negative control, and F_sample_ is the intracellular fluorescence intensity of the sample. The effect fraction (*f*a) at each drug dose was calculated.

Subsequently, the D_m_ values of the four drugs were evaluated according to the Chou-Talalay median-effect principle as follows:

2 $$\:{f}_{a}/{f}_{u}={(D/{D}_{m})}^{m}$$

Where D represents the treated dose and D_m_ denotes the dose required to produce the median-effect (e.g., 50% inhibition of ROS generation, corresponding to IC50), and m is the Hill-type coefficient, which signifies the shape of the dose-effect curve. Where *fa* represents the fraction of cells inhibiting ROS, while *fu* represents the fraction of cells not inhibiting ROS production.

On the basis of the logarithmic form of the median-effect equation:

3 $$\:\mathrm{l}\mathrm{o}\mathrm{g}({f}_{a}/(1-{f}_{a}\left)\right)=m\bullet\:\mathrm{l}\mathrm{o}\mathrm{g}\left(D\right)-\:m\bullet\:\mathrm{l}\mathrm{o}\mathrm{g}\left({D}_{m}\right)$$

Subsequently, the above equation could be transformed into a linear form as following:

4 $$\:y=m\bullet\:x+b$$

where x = log(D), y = log(*f*a/(1-*f*a)), b = -mlog(D_m_), and m is the slope. The slope is obtained by fitting each point to the linear regression equation. Consequently, the value of D_m_ is derived by transforming the intercept according to the equation [20].

### Preparation and characterization of quaternary SSPH-MPN

#### Molecular dynamics simulation for the formation of SSPH-MPN

The molecular dynamics simulation of SSPH-MPN was performed by using Materials Studio 2019. It was conducted at 1 ns. The general force field of COMPASS II was employed in molecules including SAA, SAB, PCA, HSYA, and Fe molecules. Besides, the Forcite module was used to optimize molecular structure and minimize the energy of the system.

#### Preparation of quaternary SSPH-MPNs

Quaternary SSPH-MPNs were formulated at 1:1:1:1, 2:1:1:1, 1:2:1:1, and 1:1:2:1 upon the D_m_ values of SAA, SAB, PCA, and HSYA. In brief, the four hydrophilic components were dissolved in PVP-K30 solution (5 mg/mL) to form the quaternary drug mixture solution. FeCl3·6H_2_O was dissolved in PVP-K30 solution to create the metal solution. Subsequently, the metal solution was added dropwise to the quaternary drug mixture solution under stirring. Following 24 h of vigorous stirring in the darkness under a nitrogen atmosphere at ambient conditions, the quaternary SSPH-MPN suspension was obtained via filtration and dialysis.

#### Characterization

DLS, TEM, ICP-MS, XPS, UV-vis, and FTIR were performed to evaluate the diameter, zeta potential, morphology, surface structure, and chemical composition of the SSPH-MPN. Briefly, the diameter and zeta potential of the quaternary SSPH-MPN were measured using a Malvern Zetasizer Nano ZS (Malvern, UK). The morphological images of SSPH-MPN were visualized with TEM (120 kV, Hitachi, Japan). The UV-vis absorption spectra of SSPH-MPN and the physical mixture of the four drugs (SSPH-PM) were measured using a UV-vis spectrophotometer (SHIMADZU, UV-2600). To detect the chemical compositions of the SSPH-MPN (Fe, C, Cl, N, O), XPS patterns (ESCALAB 250XI, Thermo Fisher Scientific, USA) were recorded with monochromatic Al Kα as the excitation source (hν = 1486.6 eV). ICP-MS (Agilent ICP-MS 7700) was used to determine the iron content of SSPH-MPN. FTIR spectra were recorded using a Thermo Fisher Scientific spectrometer. Furthermore, the entrapment efficiency (EE) and drug loading (DL) were determined using an HPLC (Agilent Technologies, USA) equipped with an ultraviolet detector operating at the wavelengths of 280 nm and 408 nm by gradient elution technique. The antioxidative abilities of SSPH-MPN in scavenging DPPH free radicals and superoxide anions were determined according to our previous report [20, 21].

#### Optimization of quaternary SSPH-MPNs via scavenging intracellular ROS

RAW264.7 cells were seeded in 12-well plates (2 × 10^5^ cells/well) and cultured for 12 h. Subsequently, the cells were exposed to varying concentrations of different SSPH-MPNs formulated with different D_m_ ratios of SAA, SAB, PCA, and HSYA at 1:1:1:1, 2:1:1:1, 1:2:1:1, and 1:1:2:1, respectively. The drugs with different ratios were tested for activity according to dose gradients, and the corresponding *f*a values were calculated. Dose-effect curves under the four ratios were plotted. Furthermore, the D_m_ values of the individual drugs were used to predict the synergistic index of combinations. The Combination Index (CI), a core parameter for evaluating interaction types (i.e., synergistic, additive, or antagonistic) between two or more drugs in combination, was applied. CI-*f*a curves for the four-drug combinations were generated using CompuSyn software.

### Targeting efficiency evaluation of the optimized quaternary SSPH-MPN

#### Cellular uptake study

RAW264.7 cells were seeded in a 12-well plates (2 × 10^5^ cells/per well) and cultured in DMEM with 10% FBS at 37 °C and 5% CO_2_ overnight. The optimized SSPH-MPN was labeled with R123 to obtain a fluorescent MPN, which was incubated with the cells for 1, 2, 4, 6, and 8 h, respectively. After the predetermined time intervals, the cells were washed three times with PBS to rinse the fluorescent MPN and then collected by centrifugation at 2000 × g for 10 min. Flow cytometry was utilized to determine the uptake of SSPH-MPN at an excitation wavelength of 488 nm and an emission wavelength of 525 nm. Furthermore, cells were cultured in confocal dishes and incubated with the R123-labeled SSPH-MPN for visualization. At the predetermined time points, the cells were rinsed with PBS and fixed with paraformaldehyde for 10 min. Afterwards, DAPI staining was performed for nuclear identification. CLSM was employed to visualize the sample.

#### Cellular trafficking pathway

RAW264.7 cells were seeded into 12-well plate and cultured for 12 h, followed by pretreatment with genistein (54 µg/mL), chlorpromazine (10 µg/mL), or amiloride (13.3 µg/mL) for 30 min, or incubation at 4 °C without any inhibitor treatment. Then the cells were washed three times with PBS and cultured with R123-labeled SSPH-MPN for another 6 h. The fluorescence intensity in the cells was measured by flow cytometry (λex = 488 nm, λem = 525 nm).

#### Intracellular distribution

RAW264.7 cells were seeded in confocal dishes (2 × 10^5^ cells/dish) and cultured overnight. Then, the cells were incubated with the R123-labeled SSPH-MPN for 3, 6, and 12 h at 37 °C. At the fixed time intervals, the cells were washed with PBS thrice, and then subsequently stained by DAPI for 10 min and LysoTrackerDND99 (300 nM) for 1 h to locate the nucleus and lysosomes, respectively. CLSM was employed to visualize the intracellular distribution of the SSPH-MPN.

#### In vivo atherosclerotic plaque targeting evaluations

To compare the plaque targeting efficacy of the MPNs, DiR was used to label the SSPH-MPN before the experiment. Atherosclerotic apoE^−/−^ mice were divided into two groups of three mice each. The groups received tail vein injections of the following DiR-labeled formulations at a dosage of 0.3 mg/kg DiR: (1) free DiR and (2) DiR-labeled SSPH-MPN. Four hours after injection, mice were first anesthetized using isoflurane inhalation to ensure they were unconscious and could not experience pain. Following anesthesia, the mice were euthanized via cervical dislocation. Throughout this procedure, every effort was made to minimize any potential suffering of the animals. After that, the aortic trees and major organs (heart, spleen, lungs, kidneys, and liver) of the mice were harvested for imaging, and the imaging was conducted using an IVIS system (Bruker, In Vivo FX PRO) utilizing an excitation wavelength of 720 nm and an emission wavelength of 790 nm.

### In vitro and in vivo pharmacodynamics evaluations for the optimized quaternary SSPH-MPNs

#### In vitro evaluations

##### Effect of the optimized quaternary SSPH-MPN on DiI-oxLDL uptake

RAW264.7 cells were seeded in 12-well plates (2 × 10^5^ cells/well) and cultured overnight, followed by treatment with 200 µg/mL of the optimized SSPH-MPN containing 40 µg/mL of DiI-oxLDL. After 24 h of incubation, the cells were harvested by three centrifuge steps (2000 × g, 10 min) followed by ice-cold PBS washes. The mean fluorescence intensity of DiI-oxLDL was determined by flow cytometry with an excitation wavelength of 549 nm and an emission wavelength of 565 nm. Free drugs including SAA, SAB, PCA, HSYA, and their physical mixture equivalent to 200 µg/mL of the optimized SSPH-MPN were tested by the same protocol for comparison.

##### Effect of the optimized quaternary SSPH-MPN on cellular cholesterol accumulations

RAW264.7 cells were seeded in 12-well plates (2 × 10^5^ cells/well) and cultured overnight, followed by treatment with 200 µg/mL of the optimized SSPH-MPN containing 40 µg/mL of oxLDL. After 24 h of incubation, the cells were washed with PBS thrice and then fixed with 4% paraformaldehyde for 30 min. Subsequently, the cells were washed with PBS and 60% isopropanol, and stained with Oil Red O working solution for 30 min in darkness. The cells were then washed with 60% isopropanol and PBS several times until the supernatant turned barely white. After that, the cells were visualized by the microscope. For comparison, free drugs including SAA, SAB, PCA, HSYA, and their physical mixture equivalent to 200 µg/mL of SSPH-MPN were tested by the same protocol. The cells incubated with oxLDL alone were set as the positive control. The intracellular lipid accumulation was quantified through integral optical density (IOD) analyzed by the Image-Pro Plus software.

##### Effect of the optimized quaternary SSPH-MPN on cholesterol efflux

RAW264.7 cells were seeded in 12-well plates (2 × 10^5^ cells/well) and cultured overnight, followed by incubation with 40 µg/mL of oxLDL and 5 µM of BODIPY-cholesterol. After 24 h of incubation, extracellular BODIPY-cholesterol was removed by washing the cells with cold PBS. Subsequently, the cells were treated with 200 µg/mL of the optimized SSPH-MPN, as well as equivalents of SAA, SAB, PCA, HSYA and the physical mixture for additional 12 h. After that, the cells were collected using a triple centrifugation (2000×g, 10 min). Flow cytometry was employed to measure the mean fluorescence intensity of intracellular BODIPY-cholesterol with the excitation and emission wavelengths set at 505 nm and 515 nm, respectively. For comparison, cells pretreated with BODIPY-cholesterol and oxLDL without any drug were used as positive control.

#### In vivo pharmacodynamics study

##### Establishment of an atherosclerosis model

The Animal Care and Use Committee of Southern Medical University approved all animal experiments and carried out in Institute of Biological and Medical Engineering, Guangdong Academy of Sciences. Male apoE^−/−^ mice, 6 weeks old, were placed on a high-fat diet comprising 21% fat and 0.15% cholesterol to induce atherosclerosis over a period of 12 weeks.

##### Effect of the optimized quaternary SSPH-MPN on aortic lesion areas

Atherosclerotic apoE^−/−^ mice were randomly allocated into four groups, each with six mice. Each group was respectively treated with saline, SSPH-PM, and SSPH-MPN via i.v. injections twice weekly at a dosage of 40 mg/kg. For comparison, normal apoE^−/−^ mice were treated with saline by the same protocol. After 12-week administration, mice were first anesthetized using isoflurane inhalation to ensure they were unconscious and could not experience pain or distress. Following anesthesia, blood samples were collected via retro-orbital bleeding, after which cervical dislocation was performed to ensure death. The aortic trees were collected and subjected to Oil Red O staining to measure aortic lesion areas. Throughout this procedure, every effort was made to minimize any potential suffering of the animals.

##### Effect of the optimized quaternary SSPH-MPN on serum lipid levels

The contents of HDL-C, LDL-C, TG, and TC in serum were determined by standard enzymatic assays following the manufacturer’s instructions (Jiancheng Bioengineering Institute).

##### Effect of the optimized quaternary SSPH-MPN on serum inflammatory factors

The contents of IL-6, IL-1β, and TNF-α in serum were measured by the ELISA kits from Bioswamp (Wuhan, China) following the manufacturer’s instructions.

##### Effect of the optimized quaternary SSPH-MPN on oxidative status of aorta tissues

To assess the in situ ROS content in the aorta, the oxidative fluorescent dye dihydroethidine (DHE) was employed to monitor the ROS in frozen aortic rings of different groups as described by Buday et al. [22]. DAPI was used to visualize the nuclei. Aortic sections were imaged by fluorescence microscopy.

##### Effect of the optimized quaternary SSPH-MPN on liver oxidative enzymes

To determine the activities of SOD, GSH-Px, MDA, and T-AOC in liver, the liver homogenates of apoE^−/−^ mice after treatment of different samples for 12 weeks were used for the analysis of the antioxidant enzymes’ activity by using the commercially available kits according to the manufacturer’s instructions (Jiancheng Bioengineering Institute, China).

### Safety evaluations of the optimized quaternary SSPH-MPN

#### Hemolysis assay

Fresh rabbit red blood cells were collected after continuous washing with PBS (pH 7.4) by centrifugation at 1000 × g for 10 min several times. Then, 2% (v/v) of red blood cells was prepared for the hemolysis test. Aliquots (750 µL) of red blood cells were added to centrifuge tubes, followed by addition of the same volume of the optimized SSPH-MPN suspension with different concentrations (25, 50, 100, 200, 300, 400 to 500 µg/mL). After incubation in a shaker at 37 °C for 3 h, the samples were centrifuged at 2000 × g for 10 min. The absorbance of the supernatant was measured at 540 nm by using a microplate reader (Biotek ELx800). PBS and H_2_O were added to the red blood cell solution for the same treatment as negative and positive controls, respectively. The hemolysis rate was calculated as follows:

5 $$\:\mathrm{H}\mathrm{e}\mathrm{m}\mathrm{o}\mathrm{l}\mathrm{y}\mathrm{s}\mathrm{i}\mathrm{s}\:\mathrm{r}\mathrm{a}\mathrm{t}\mathrm{e}\left(\%\right)=\frac{{A}_{sample}-{A}_{negative\:control}}{{A}_{positive\:control}-{A}_{negative\:control}}\times\:100\%$$

#### Cytotoxicity study

RAW 264.7 cells were seeded into 96-well plates at a density of 1 × 10^4^ cells per well and incubated at 37 °C with 5% CO_2_ in DMEM supplemented with 10% FBS for 12 h. Then, different concentrations of the optimized SSPH-MPN suspension were added into the cells (25, 50, 100, 200, 300, 400 to 500 µg/mL). Following a 24 h treatment, the cells underwent three washes and then treated with MTT solution (200 µL, 0.5 mg/µL). After 4 h of incubation, the MTT solution was removed, and 150 µL of DMSO was added to solubilize the formazan crystals. The optical absorbance value was recorded with a microplate reader at 570 nm.

#### In vivo safety tests

Atherosclerotic apoE^−/−^ mice were administered two i.v. injections of SSPH-MPN per week for 12 weeks. The atherosclerotic apoE^−/−^ mice and apoE^−/−^ receiving saline following the same dosing regimen were set as positive and negative control groups, respectively. After administration for 3 months, the blood sample was collected for biochemical analysis of ALT and AST by commercial kits obtained from Jiancheng Bioengineering Institute (Nanjing, China) following the manufacturer’s instructions.

### Statistical analyses

Data were presented as mean ± standard deviation (SD). Statistical analysis was conducted with SPSS by using Student’s t-test for two groups and ANOVA for multiple groups. Significance was reported as *p < 0.05, **p < 0.01.

## Results and discussions

### Machine learning-aided hybrid network pharmacology-QSAR method identified SAA, SAB, PCA, and HSYA as the core components in *Salvia miltiorrhiza* and *Carthamus tinctorius*

**Fig. 1:**
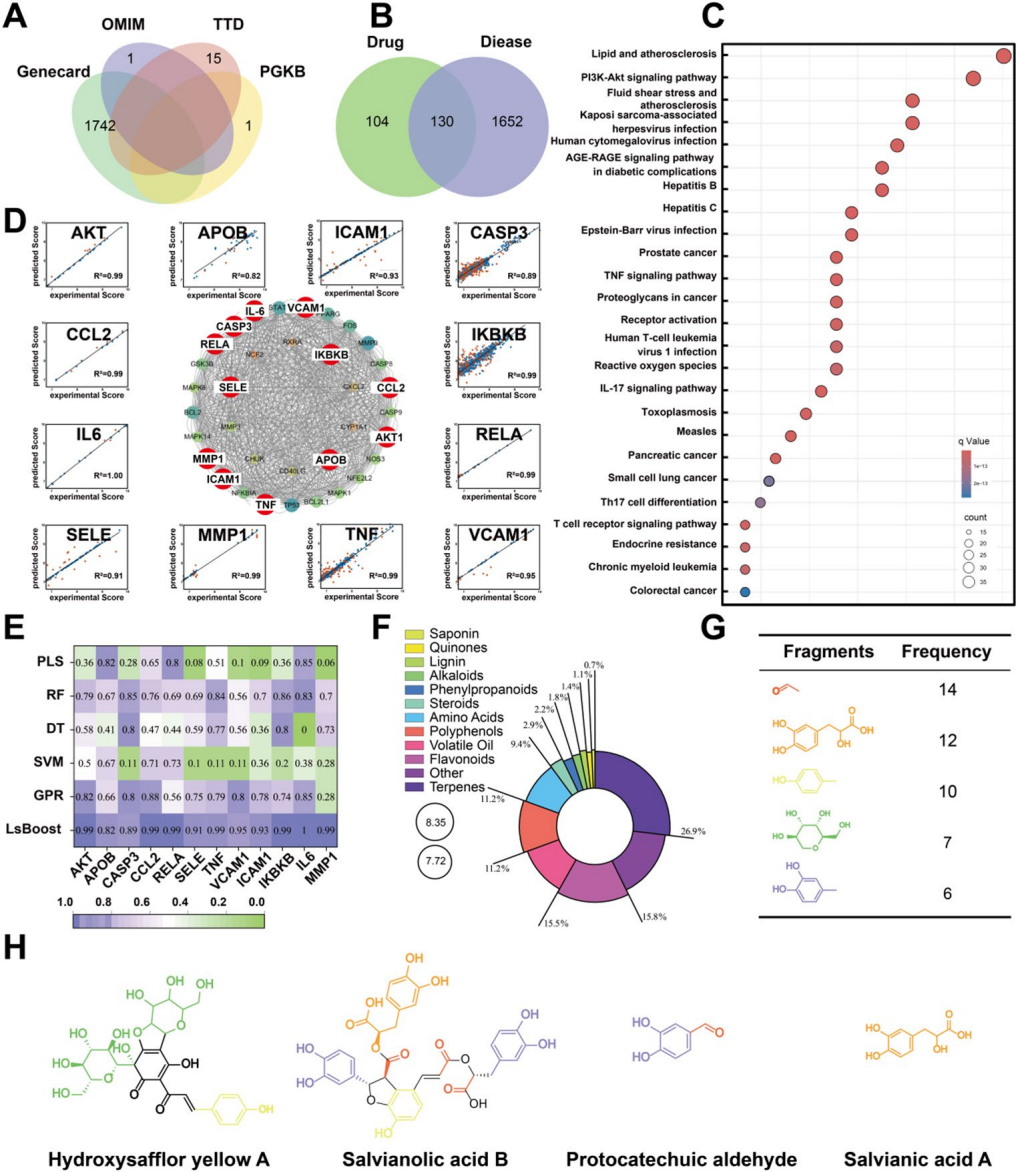
Screening of key components. **(A)** Venn diagram of disease-related targets. **(B)** Venn diagram of shared targets between AS disease and herbal drugs. **(C)** KEGG pathway enrichment analysis. **(D)** PPI network of the most correlated pathways and machine learning-based QSAR model performance of the targets in the pathway. **(E)** Performance of various machine learning algorithms in fitting QSAR models. **(F)** Proportional representation of the components and their average prediction scores, with the size of each circle representing the score. **(G)** Fragment analysis of the top 50 scoring components. **(H)** Chemical structures of the key components

Network pharmacology was employed to identify the major pathways involved in the treatment of AS by *Salvia miltiorrhiza* and *Carthamus tinctorius*. Initially, 1,769 disease-related target genes for AS were collected from open-source databases (Fig. 1A). Therapeutic targets of traditional Chinese medicine were extracted from the TCMSP database, and the cross-targets of disease-related genes revealed 130 shared genes (Fig. 1B). KEGG pathway enrichment analysis of the intersecting genes identified the top three pathways as “Lipids and Atherosclerosis”, “PI3K-Akt signaling pathway” and “Fluid shear stress and Atherosclerosis” (Fig. 1C), all of which are closely related to the pharmacological effects of the two TCM, such as blood lipid reduction, lipid metabolism regulation, and vasodilation [23, 24]. According to the ranking based on the smallest q-value, the pathways most associated with atherosclerosis were described as “Lipid and atherosclerosis” (Table S1). Among these, 35 target genes were common to both the drug and disease. Additionally, PPI network analysis using the STRING database showed a strong correlation among these genes, with an MCODE score of 11.8. To construct a QSAR model for these genes, various machine learning methods were tested, with description and parameter settings provided in Table S2. To assess the validity of our descriptor selection and train-test partitioning strategy, t-SNE dimensionality reduction was performed on all 12 target proteins (Fig. S1). The visualization reveals a uniform distribution of samples across both training and testing sets under the current feature space, substantiating the robustness of our data splitting approach. Among various machine learning models, the LsBoost algorithm exhibited the best predictive performance, significantly outperforming other algorithms in fitting different QSAR models. Hyperparameter optimization systematically explored the configuration space of MinLeafSize, NumCycles, and LearningRate parameters (Fig. S2). Hot regions representing the top 20% performing configurations were identified for targeted analysis. Performance sensitivity analysis across six MinLeafSize values (1–20) revealed distinct optimal zones (Fig. S3). MinLeafSize = 10 achieved the highest Stage 1 performance (R^2^ = 0.9705) at a learning rate 0.5 and 200 cycles. Smaller MinLeafSize values exhibited broader optimal regions, while larger values showed narrower peaks with reduced accuracy. Comparative analysis demonstrated substantial improvements over the baseline (Fig. S4). The default configuration (NC = 10, LR = 1.0, ML = 1) yielded *R*^2^ = 0.6564. The Stage 1 best configuration remarkably improved performance (R^2^ = 0.9705), while the optimized configuration (NC = 200, LR = 0.50, ML = 10) achieved the highest accuracy (R^2^ = 0.9822), representing a 49.63% improvement over the baseline. Computational cost analysis revealed a linear relationship between training cycles and execution time (Fig. S5), with training time increasing from 0.4 s at 100 cycles to 1.5 s at 300 cycles. The optimal configuration maintained maximum performance while preserving computational efficiency.

Therefore, this algorithm was applied to develop a QSAR model for the genes in the “Lipid and Atherosclerosis” pathway. A search for real experimental data of small molecules in the ChEMBL database identified corresponding records for 33 out of the 35 targets, which are summarized in Table S3. Furthermore, activity types contributing to AS treatment were selected based on a literature review, such as IC50 for the APOB protein, which plays a critical role in lowering LDL levels and preventing the progression of AS [25]. After excluding datasets with fewer than 28 samples, the remaining 12 target datasets were used for QSAR model construction (Fig. 1D). These LsBoost algorithm-based models exhibited excellent precision (0.82 ~ 1.00) and demonstrated robust predictive potential as evidenced by the similar MSE, MAE, RMSE, and R^2^ values between training and validation sets (Table S4). Feature importance analysis revealed the top 10 contributing descriptors for each target protein based on LsBoost intrinsic scoring metrics (Fig. S6). The QSAR models were then used to predict all compounds derived from the two herbs. In terms of compound classification, terpenes, flavonoids, and polyphenols represented 26.9%, 15.8%, and 11.2%, respectively (Fig. 1E).

The radius of the pie chart represents the prediction score, based on the average of the 12 models’ predictions, with a ten-point scoring system. Among the compounds, flavonoids had the highest average score of 8.27, followed by polyphenols with a score of 8.24 (Fig. 1F). To further refine the selection of core ingredients, the top 50 compounds were subjected to molecular docking (the proteins used for docking are listed in Table S5). The docking results (Table S6), combined with QSAR predictions, identified the top five compounds as lignan, SAB, Salvianolic acid E, Dihydroisotanshinone I, and HSYA. Among them, SAB and HSYA are recognized as standard markers for *Salvia miltiorrhiza* and *Carthamus tinctorius*, as evidenced by the 2025 edition of the Chinese Pharmacopoeia [26]. Molecular fragment analysis of the top 50 compounds revealed that SAA appeared 12 times, highlighting the importance of this structure (Fig. 1G). Similarly, the PCA showed significant overlap with these fragments. Taken together, SAA, SAB, PCA, and HSYA were selected as core ingredients for *Salvia miltiorrhiza* and *Carthamus tinctorius* (Fig. 1H).

### Optimization and characterization of the optimized quaternary SSPH-MPN

**Fig. 2:**
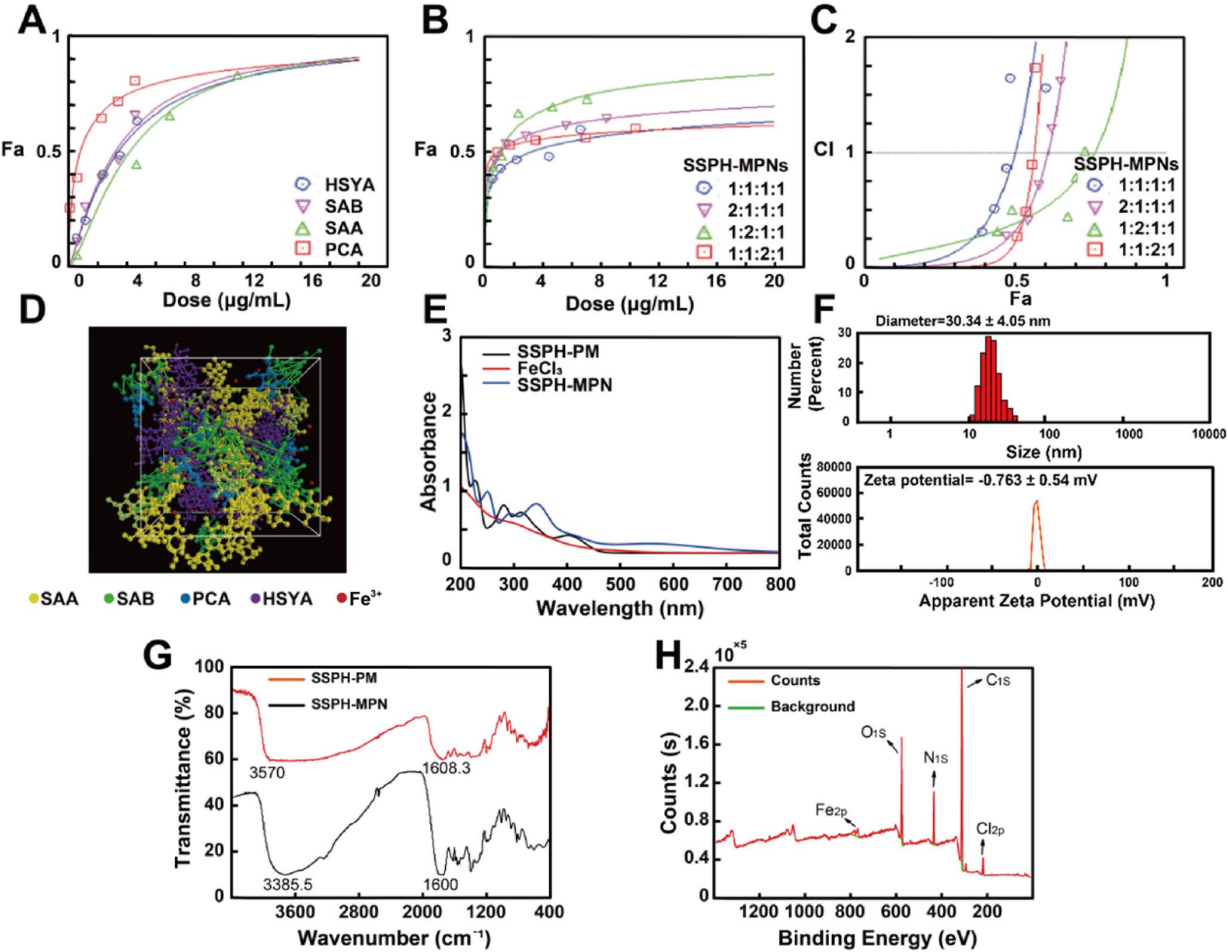
Optimization and characterization of quaternary SSPH-MPN. **(A)** Dose-effect relationship curves of SAA, SAB, PCA, and HSYA. **(B)** Dose-effect relationship curves of different SSPH-MPN formulated with 1:1:1:1, 2:1:1:1, 1:2:1:1, 1:1:2:1 (D_mSAA_: D_mSAB_: D_mPCA_: D_mHSYA_). **(C)** CI-Fa curves of different SSPH-MPN formulated with 1:1:1:1, 2:1:1:1, 1:2:1:1, 1:1:2:1 (D_mSAA_: D_mSAB_: D_mPCA_: D_mHSYA_). **(D)** The molecular dynamics simulation of the SSPH-MPN. Yellow: SAA, green: SAB, blue: PCA, purple: HSYA, red: Fe^3+^. **(E)** UV-vis spectra of SSPH-PM, FeCl₃, and SSPH-MPN. **(F)** Particle size and zeta potential of SSPH-MPN. **(G)** FTIR spectra of SSPH-PM and SSPH-MPN. **(H)** XPS spectrum of SSPH-MPN

Based on the polyphenolic structure of the selected ingredients, we aimed to construct an MPN-based system for the co-delivery of the four components via coordination. Initially, we screened the optimized synergistic ratio of the four components using the median-effect principle. Given that the selected polyphenolic components exhibit potent antioxidative potential and oxidative stress is a key early driver of AS [27], intracellular ROS scavenging was chosen as the efficacy indicator for optimization. We established dose-effect relationships for the four active components (SAA, SAB, PCA, and HSYA) based on the median-effect principle to determine their median-effect doses (D_m_). Figure 2A illustrates the dose-effect curves, and their respective D_m_ values were calculated as 4.21, 3.20, 0.78, and 3.42 µg/mL (Table 1). PCA exhibited the strongest ROS scavenging activity due to the lowest D_m_ value, while SAA showed the weakest activity (highest D_m_ value) at equivalent concentrations.

**Table 1:** Dose-effect parameters of SAA, SAB, PCA, HSYA, and different quaternary SSPH-MPN with the effect on scavenging intracellular ROS in RAW264.7 cells (*n* = 3)

Tested components/formulations		D_m_ (µg/mL)^a^	*r*
SAA		4.21	0.98
SAB		3.20	0.98
PCA		0.78	0.98
HSYA		3.42	0.99
SSPH-MPNs^b^	1:1:1:1	2.93	0.92
	2:1:1:1	0.97	0.99
	1:2:1:1	0.94	0.97
	1:1:2:1	0.74	0.96

^a^D_m_ is the median-effect dose (concentration that scavenges intracellular ROS by 50%), r is the linear correlation coefficient of the median-effect curve

^b^SSPH-MPNs were formulated with different ratios of SAA, SAB, PCA, and HSYA based on their D_m_ values

Subsequently, four SSPH-MPN formulated with ratios of 1:1:1:1, 2:1:1:1, 1:2:1:1, and 1:1:2:1 (based on the D_m_ ratios of SAA, SAB, PCA, and HSYA) were prepared. Dose-effect relationships for the SSPH-MPN were established (Fig. 2B), revealing D_m_ values of 2.93, 0.97, 0.94, and 0.74 µg/mL, respectively (Table 1). The SSPH-MPN formulated with 1:1:2:1 D_m_ ratio exhibited the most profound ROS scavenging effect at concentrations below 0.74 µg/mL, likely due to the higher proportion of PCA in this formulation. Compared to the individual components, the D_m_ values of the SSPH-MPN were remarkably reduced, indicating that the MPN-based delivery system enhanced the antioxidative effect of the hydrophilic polyphenols. To identify the optimal formulation, we plotted combination index (CI)-Fa curves for the four SSPH-MPN using CompuSyn software (Fig. 2C). The results showed that CI values increased with Fa levels, indicating synergism at low Fa levels and antagonism at high Fa levels. Among the four SSPH-MPN, the 1:2:1:1 formulation had the widest region with CI values below 1, suggesting the broadest synergistic effect. Therefore, the SSPH-MPN formulated with a 1:2:1:1 D_m_ ratio was selected as the optimized formulation for further study.

Molecular dynamics simulation was performed to simulate the construction of SSPH-MPN using Material Studio 2019 software. As shown in Fig. 2D, the quaternary system exhibited aggregated distributions, suggesting a strong potential for the assembly of the four components with iron ions. The optimized SSPH-MPN was characterized by a series of studies, including UV-vis, DLS, FTIR, XPS, and ICP-MS. It was found that the UV-vis spectrum of the SSPH mixture solution showed several maximum absorbance peaks at λ = 227, 281, 313, and 402 nm. However, after the addition of iron ions, the UV-vis spectrum of SSPH-MPN showed prominent bathochromic shifts to 250, 293, and 340 nm. Interestingly, the characteristic absorbance peak of HSYA at 402 nm completely disappeared, but the broad characteristic ligand-to-metal charge transfer band of catechol moieties interacting with iron ions located at 555 nm appeared (Fig. 2E), probably indicating the assembly of SSPH-MPN [28, 29]. DLS illustrated that the particle size and zeta potential of SSPH-MPN suspension were 30.34 ± 4.05 nm and − 0.763 ± 0.54 mV, respectively (Fig. 2F). To further confirm the successful assembly of quaternary SSPH-MPN, the suspension was lyophilized. FTIR spectrum of the SSPH-PM exhibited an intense absorption close to 3570 cm^− 1^ (Fig. 2G), corresponding to the stretching mode of hydroxyl groups [30]. After chelation with iron ions, the absorption band was broadened and showed a prominent hypochromatic shift to 3385.5 cm^− 1^. Similarly, the stretching mode of carbonyls of the SSPH-MPN was shifted from 1608.3 cm^− 1^ to 1600 cm^− 1^, which demonstrates that the carbonyl hydroxyl groups might also participate in the formation of coordination bond with the iron ion [31]. To monitor the elemental compositions of the quaternary SSPH-MPN, an XPS spectrum was recorded. We observed that the SSPH-MPN contains multiple elements, including O, N, C, and Cl as evidenced by the four strong peaks at binding energies of ~ 531, 400, 284, and 197 eV, respectively (Fig. 2H). Moreover, the iron ions in the SSPH-MPN mainly existed as Fe^III^ due to the appearance of the Fe 2p peaks at binding energies of ~ 710 and 724 eV [32]. The content of iron ion was approximately 49.5 mg/g as measured by ICP-MS, further proving the successful preparation of the quaternary MPN [33].

### The optimized quaternary SSPH-MPN was primarily internalized through clathrin-mediated endocytosis and demonstrated a good plaque targeting efficiency

**Fig. 3:**
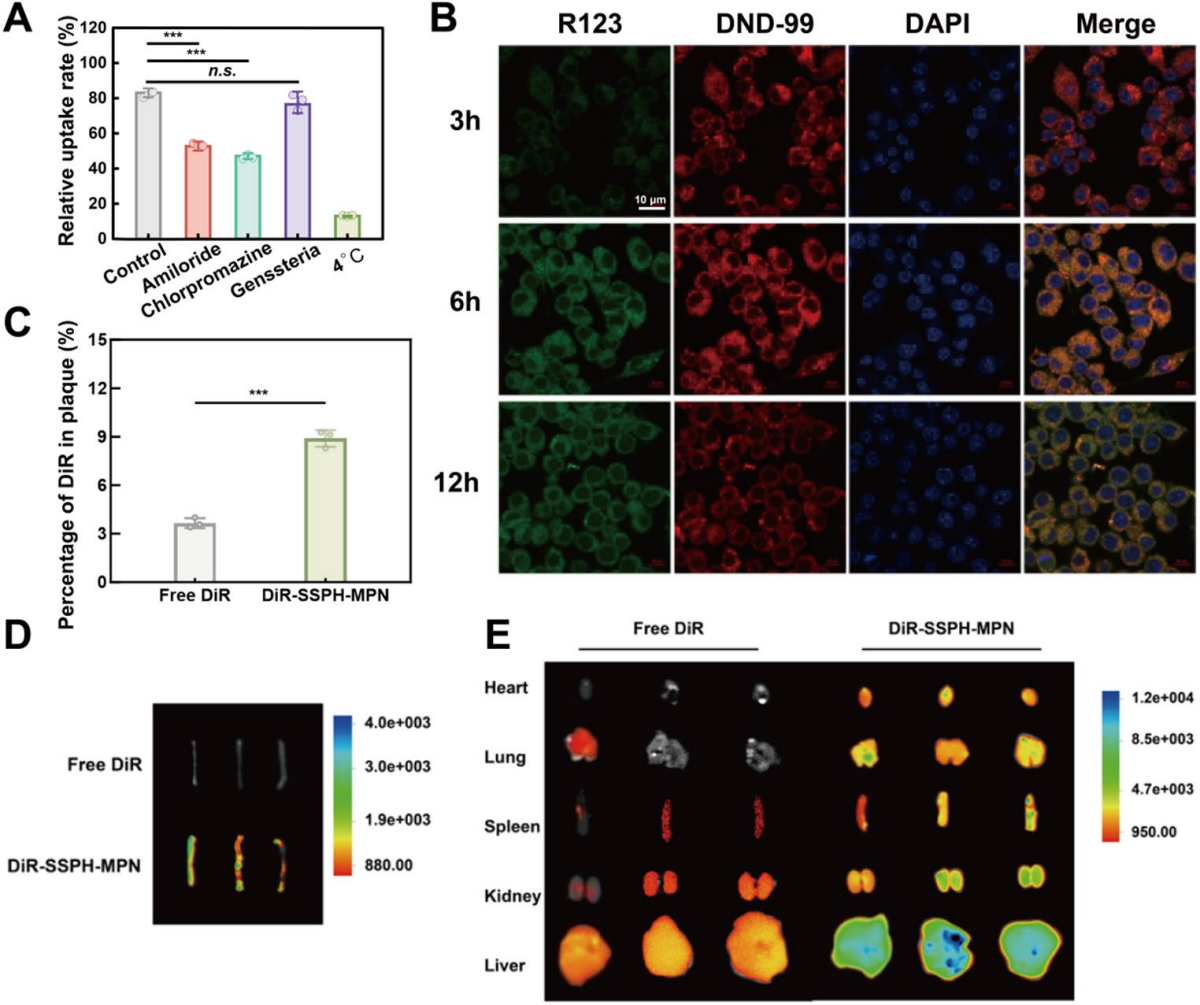
In vitro and in vivo targeting capability evaluations of SSPH-MPN. **(A)** Effect of different inhibitors on the cellular uptake of R123-SSPH-MPN (*n* = 3, ***p < 0.001). **(B)** Subcellular distribution of R123-SSPH-MPN. Late endolysosomes were stained with LysoTracker Red DND-99, nuclei were stained with DAPI, and green fluorescence indicates R123-SSPH-MPN. Scale bar: 10 μm. **(C)** Plaque accumulation analysis of free DiR and DiR-SSPH-MPN group in atherosclerotic apoE^−/−^ mice following tail vein injection for 4 h (*n* = 3, ***p < 0.001). **(D)** Ex vivo fluorescence imaging of aortic trees and **(E)** major organs in atherosclerotic apoE^−/−^ mice after treatment of free DiR and DiR-SSPH-MPN

To monitor the cellular uptake behavior of the optimized SSPH-MPN, fluorescent R123 was used to label the SSPH-MPN. Figure S7A illustrates the cellular uptake kinetics of R123-SSPH-MPN. The mean fluorescence intensity gradually increased with time, reaching a plateau at 6 h, suggesting that the cellular uptake of the SSPH-MPN was saturated by this time. To visualize the cellular uptake behavior, CLSM was employed. Consistent with the flow cytometry results, the intracellular fluorescence signal of SSPH-MPN increased over time, peaking at 6 h (Fig. S7B), further indicating that the cellular uptake saturated at this point. To investigate the cellular uptake mechanism of SSPH-MPN, cells were initially incubated at 4 °C, revealing that endocytosis is energy-dependent, as cellular uptake decreased to 12.9% at 4 °C compared to the control group (Fig. 3A). To further explore the cellular uptake mechanisms of SSPH-MPN, the cells were pretreated with three types of endocytosis inhibitors (amiloride, chlorpromazine hydrochloride, and genistein). Amiloride, an inhibitor of macropinocytosis, caused a 36.9% reduction in the cellular uptake of SSPH-MPN. A further decrease (~ 43.3%) was observed following pretreatment with chlorpromazine hydrochloride, suggesting that clathrin-mediated endocytosis plays a significant role in the cellular internalization of SSPH-MPN. Notably, genistein had little effect on the cellular uptake of SSPH-MPN, indicating that caveolae-mediated endocytosis is not involved in the internalization process. Subsequently, we examined the intracellular distribution of R123-SSPH-MPN using CLSM to further elucidate its trafficking mechanism. As shown in Fig. 3B, the intracellular fluorescence signal of SSPH-MPN was faint at 3 h but intensified by 6 h, indicating saturation of SSPH-MPN uptake. The merged fluorescence signals of R123 (green) and LysoTracker DND-99 (red) revealed that SSPH-MPN localized within endolysosomes, which was consistent with the flow cytometry data. After 12 h of incubation, partial separation of R123 and DND-99 signals suggested that some SSPH-MPN escaped from endolysosomes, further supporting that clathrin-mediated endocytosis as the primary cellular entry mechanism for SSPH-MPN [34].

To validate the in vivo targeting efficacy of SSPH-MPN for atherosclerotic plaques, DiR-labeled SSPH-MPN was used to monitor its in vivo distribution in atherosclerotic apoE^−/−^ mice. As shown in Fig. 3D-E, minimal fluorescence was detected in the aortic tree, with most fluorescence accumulating in the lung and liver for the free DiR group. In contrast, obvious fluorescence was observed in the aortic tree of the DiR-SSPH-MPN group. Based on the fluorescence ratio calculated as the fluorescence intensity in plaques divided by the sum of fluorescence intensities in major organs and plaques (Table S7), we found that the percentage of DiR-SSPH-MPN accumulated in the plaque was approximately 2.43-fold higher than that in the free DiR group (Fig. 3C), suggesting that SSPH-MPN is capable of co-delivering the four core components to atherosclerotic plaques.

### The optimized quaternary SSPH-MPN exhibited greater efficiency in inhibiting lipid uptake, mediating cholesterol efflux, and attenuating oxidative stress than SSPH-PM in RAW264.7 cells

**Fig. 4:**
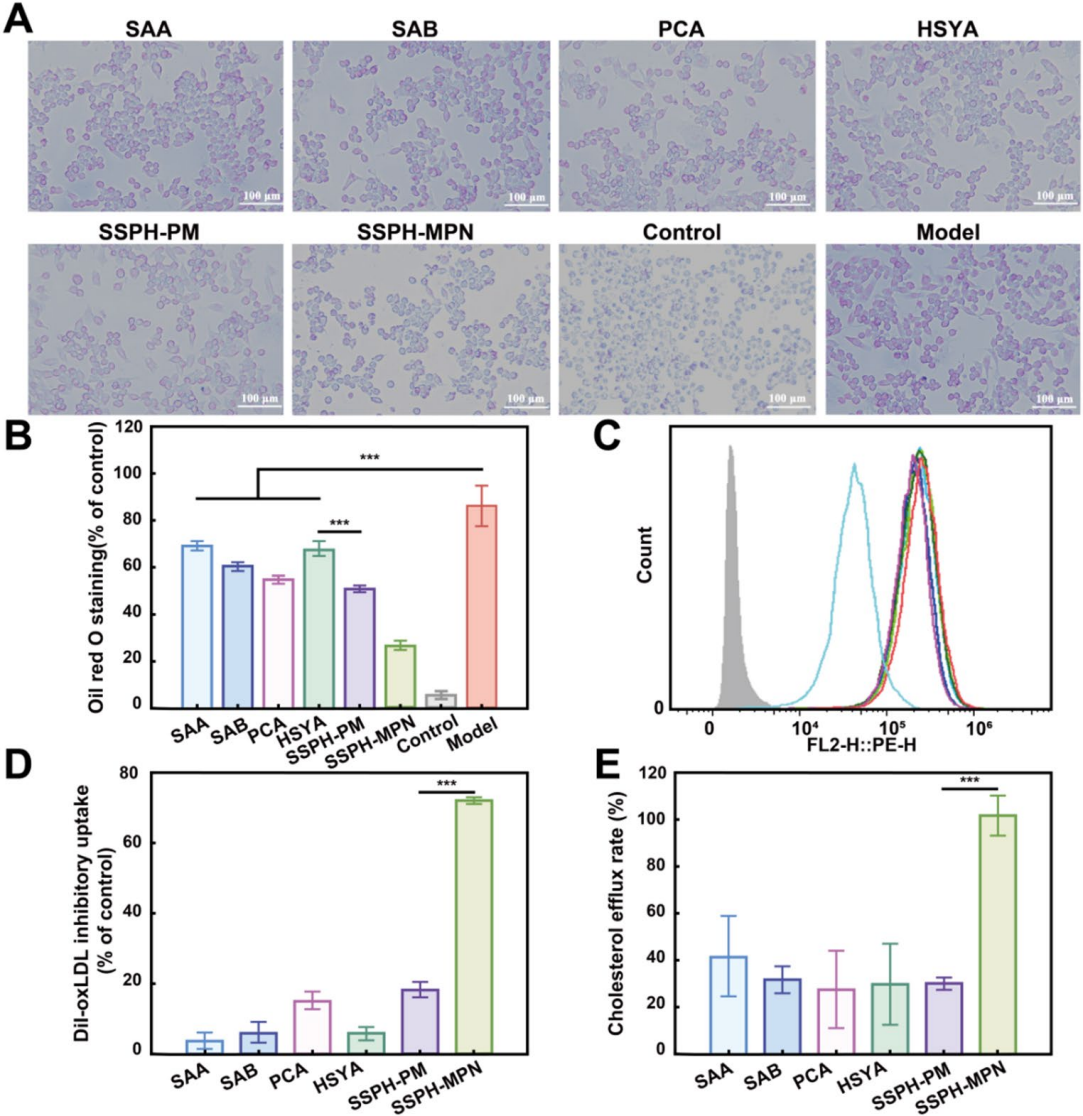
In vitro pharmacodynamics evaluations of the optimized SSPH-MPN. **(A)** Representative images of Oil Red O staining for the optimized SSPH-MPN at a concentration of 200 µg/mL and the equivalent amounts of SAA, SAB, PCA, HSYA, and SSPH-PM. Scale bar: 100 μm. **(B)** Quantitative data of Oil Red O staining for the optimized SSPH-MPN at the same concentration and equivalent amounts of SAA, SAB, PCA, HSYA, and SSPH-PM (*n* = 3, ***p < 0.001). **(C)** Inhibitory uptake rates of DiI-oxLDL by the optimized SSPH-MPN at a concentration of 200 µg/mL and the equivalent amounts of SAA, SAB, PCA, HSYA, and SSPH-PM. **(D)** Flow cytometry analysis of DiI-oxLDL uptake following internalization of the different samples (*n* = 3, ***p < 0.001). **(E)** BODIPY-cholesterol efflux rates of the optimized SSPH-MPN at the concentration of 200 µg/mL and the equivalent amounts of SAA, SAB, PCA, HSYA, and SSPH-PM (*n* = 3, ***p < 0.001)

To detect intracellular lipid deposition, Oil Red O staining was performed. As shown in Fig. 4A, large red deposits were observed in the positive control group. In contrast, the area of Oil Red O staining gradually decreased in the following order after treatment with HSYA, SAB, PCA, SAA, SSPH-PM, and SSPH-MPN. Compared to the positive control group, SAA, SAB, PCA, and HSYA exhibited significant lipid deposition, ranging from 61.0% to 78.8% (Fig. 4B). Less lipid deposition was observed for the physical mixture of the four components compared to the positive control group (~ 56.7%), suggesting that the four active compounds from *Salvia miltiorrhiza* and *Carthamus tinctorius* may exert a synergistic effect. Notably, among the six samples, the optimized SSPH-MPN exhibited the lowest staining area, showing approximately a 73.6% reduction compared to the positive control. This result demonstrates the great potential of MPN-based nanoparticles in enhancing the therapeutic efficacy of the drugs. Increasing evidence suggests that cellular lipid accumulation is primarily due to increased lipid uptake and reduced cholesterol efflux [35, 36]. Therefore, we conducted DiI-oxLDL uptake and cholesterol efflux studies. As shown in Fig. 4C-D, SAA, SAB, and HSYA exhibited weak inhibitory effects on DiI-oxLDL uptake, with reductions ranging from approximately 4.0% to 6.4%. Among them, PCA displayed the most potent inhibitory effect (~ 16.3%). A further inhibition (~ 20.5%) was achieved with the SSPH-PM. Impressively, the inhibition of DiI-oxLDL uptake was markedly enhanced after treatment with SSPH-MPN, resulting in approximately a 3.9-fold increase. This could be one of the key factors contributing to the remarkable reduction in lipid deposition observed with SSPH-MPN. Subsequently, their cholesterol efflux capability was explored by detecting the intracellular BODIPY-cholesterol content. Different from the inhibitory effect on DiI-oxLDL uptake, the cholesterol efflux rates were reduced after treatment with six samples. We found SAA, SAB, HSYA, and their physical mixture had a weak effect on mediating cholesterol efflux from lipid-laden macrophages in the range of approximately 3.1–7.3% (Fig. 4E). Among them, the effect of SAA was the highest. As expected, the optimized SSPH-MPN showed the most prominent effect on mediating cholesterol efflux, achieving approximately 3.4-fold enhancement compared with SAA, which further results in the reduction in lipid accumulation.

### The optimized quaternary SSPH-MPN exhibited potent anti-atherosclerotic efficacy in apoE^−/−^ mice

**Fig. 5:**
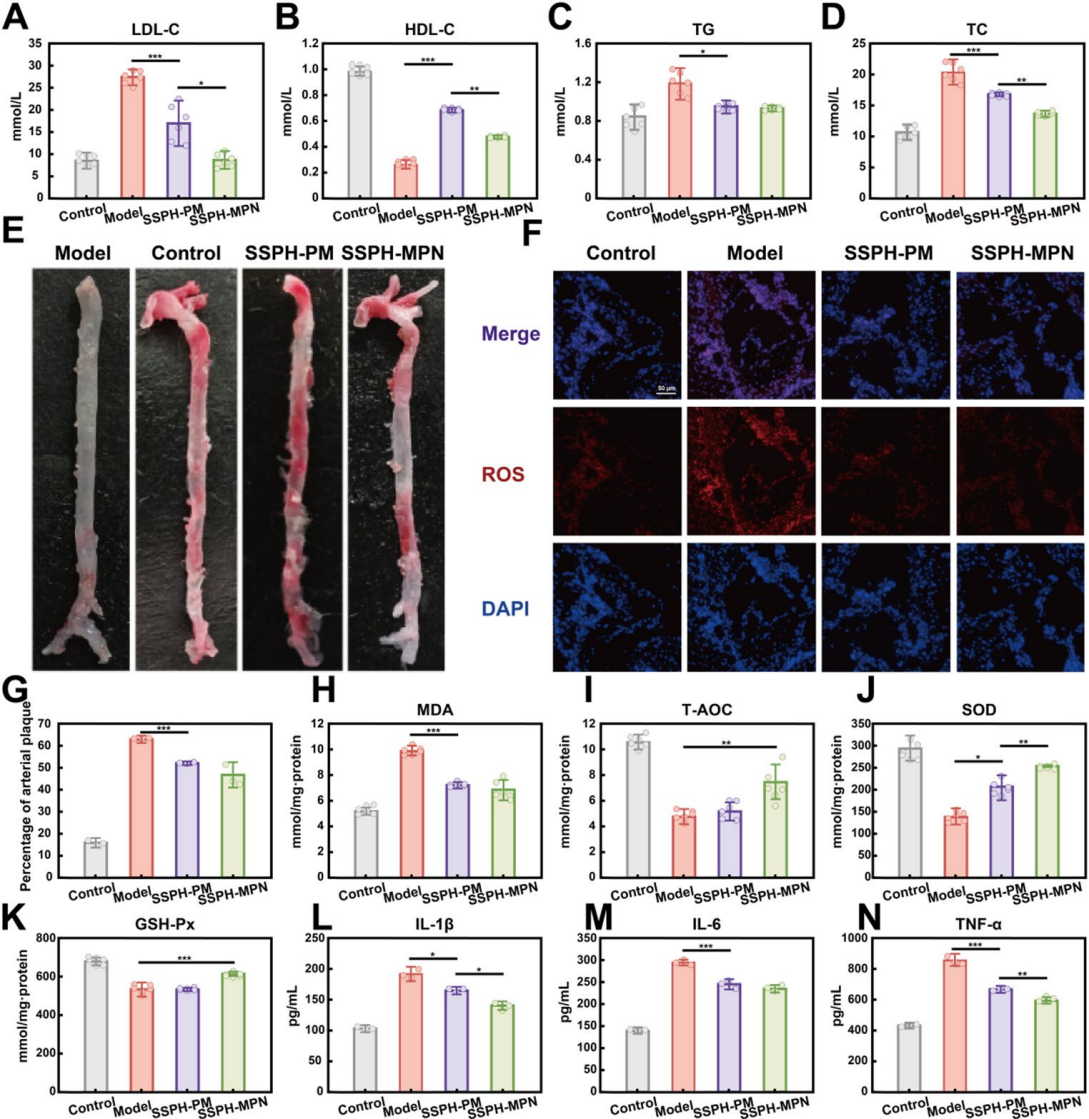
In vivo pharmacodynamic evaluation. Serum lipid levels of (**A**) LDL-C, (**B**) HDL-C, (**C**) TG and (**D**) TC in apoE^−/−^ mice treated with different samples (*n* = 3, *p < 0.05, ***p < 0.001). **(E)** Representative images of aortic roots stained with Oil Red O in apoE^−/−^ mice treated with different samples. **(F)** Representative images showing ROS in aortas measured by DHE staining after treatment of different samples. Blue represents the nucleus, and red indicates the DHE probe (Scale bar: 50 μm). **(G)** The proportion of aortic plaque determined by the results of Oil Red O staining (*n* = 3, ***p < 0.001). Liver antioxidant enzyme activities of **(H)** MDA and **(I)** T-AOC, **(J)** SOD and **(K)** GSH-Px (*n* = 3, *p < 0.05, **p < 0.01, ***p < 0.001). Serum levels of the inflammatory cytokine **(L)** IL-1β, **(M)** IL-6, and **(N)** TNF-α following treatment with different samples (*n* = 3, *p < 0.05, **p < 0.01, ***p < 0.001)

To evaluate the in vivo anti-AS effects of SSPH-MPN, serum lipid levels were assessed. As shown in Fig. 5A-D, the levels of TC and LDL-C were significantly higher in the model group compared to the negative control group. After SSPH-MPN treatment, the levels of TC, TG, and LDL-C were remarkably reduced, while the level of HDL-C was significantly higher in the SSPH-MPN group (*p < 0.001*). These changes in lipid levels suggest that the SSPH-MPN may help reduce atherosclerotic plaque accumulation. To confirm this, aortic blood vessels were stained with Oil Red O solution. As shown in Fig. 5E, minimal plaque was observed in the negative control group, while significant atherosclerotic plaque was present in the model group. Treatment with SSPH-MPN led to a 30.0% reduction in plaque area compared to the model group (Fig. 5G). Given that oxidative stress is a pivotal factor in the pathogenesis of AS, we assessed the anti-oxidative properties of the optimized SSPH-MPN. The in vitro antioxidative assays, including the DPPH radical scavenging test and the superoxide anion scavenging test, indicated that the optimized SSPH-MPN possesses a significant scavenging capacity that intensifies with increasing concentration (Fig. S8). For in vivo antioxidant activity, we visualized reactive oxygen species (ROS) content in the aorta using the DHE probe. CLSM imaging revealed that DHE fluorescence in the SSPH-MPN group was notably weaker than in the model group, similar to the negative control, suggesting that the SSPH-MPN efficiently clears ROS in the aorta (Fig. 5F**)**. The ROS levels in atherosclerotic plaques might be alleviated by the enhanced antioxidant enzyme activity. To confirm this, we evaluated the effects of SSPH-MPN on liver antioxidant enzyme activities. As shown in Fig. 5H-K, the activities of superoxide dismutase (SOD) and total antioxidant capacity (T-AOC) were significantly higher in the SSPH-MPN group compared to the saline-treated model group (*p < 0.01*), while malondialdehyde (MDA) activity was significantly lower (*p < 0.001*). These findings indicate that the MPN-based delivery system effectively enhances liver antioxidant enzyme activity. Overall, these results demonstrate that SSPH-MPN exhibits potent antioxidative activity. Moreover, the inhibitory effects of SSPH-MPN on serum inflammatory cytokines IL-6, IL-1β, and TNF-α were evaluated. In the model group, secretion of these cytokines was significantly increased compared to the negative control. After SSPH-MPN treatment, a potent inhibitory effect on IL-1β, IL-6, and TNF-α secretion was observed, with reductions of 26.7%, 20.4%, and 26.9%, respectively, compared to the model group (Fig. 5L-N).

### SSPH-MPN displayed good biocompatibility

**Fig. 6:**
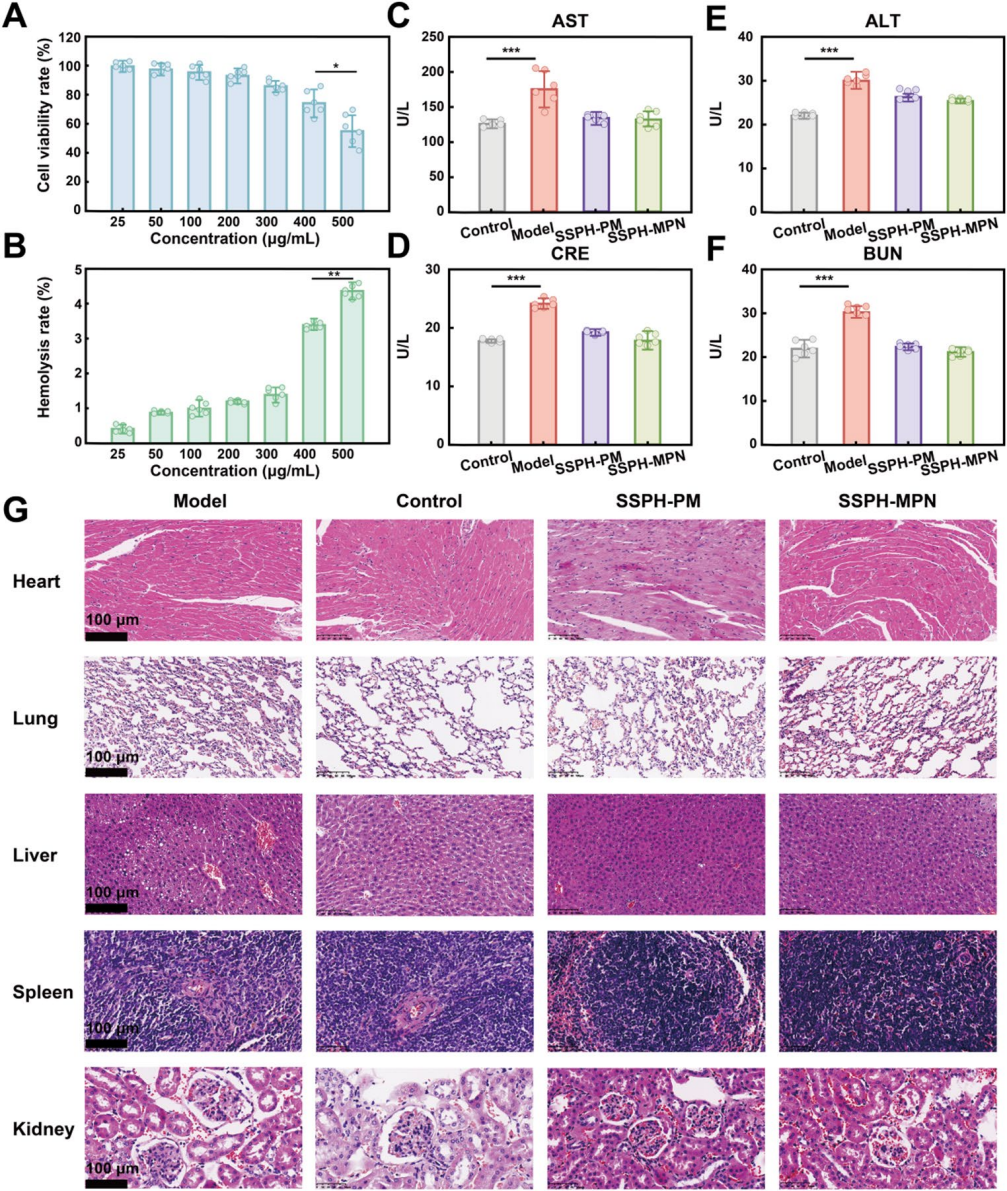
In vitro and in vivo safety evaluation. **(A)** Cell viability rates of SSPH-MPN at the concentrations of 25, 50, 100, 200, 300, 400 to 500 µg/mL (*n* = 6, *p < 0.05). **(B)** Hemolysis rates of SSPH-MPN at the concentrations of 25, 50, 100, 200, 300, 400 to 500 µg/mL (*n* = 6, **p < 0.01). Activities of **(C)** AST and **(D)** CRE, **(E)** ALT and **(F)** BUN of the atherosclerotic apoE^−/−^ mice in the treatment of SSPH-PM, SSPH-MPN or saline (*n* = 3, ***p < 0.001). **(G)** H&E histopathological staining of major organs collected from atherosclerotic apoE^−/−^ mice in the treatment of SSPH-PM, SSPH-MPN or saline. Scale bar: 100 μm

Good biocompatibility is a fundamental requirement for an ideal drug delivery carrier. In this study, the biocompatibility of the optimized SSPH-MPN was evaluated through hemolysis, cytotoxicity, and in vivo safety tests. To assess the cytotoxicity of SSPH-MPN, an MTT assay was conducted. We found that SSPH-MPN exhibited high cell viability at concentrations up to 200 µg/mL (Fig. 6A). However, significant cell toxicity was observed at higher concentrations. Cell viability significantly decreased at 400 µg/mL (*p < 0.05*), and further reduction was noted at 500 µg/mL. As shown in Fig. 6B, we observed that the hemolysis rate increased with higher concentrations of SSPH-MPN. The hemolysis rate significantly increased when the concentration of SSPH-MPN exceeded 400 µg/mL (*p < 0.01*). Despite the increase in hemolysis, the rate remained below the internationally recognized threshold (5%) for red blood cell damage, suggesting that SSPH-MPN is biocompatible at concentrations ranging from 25 to 500 µg/mL. Additionally, the long-term (3-month) safety of the optimized SSPH-MPN was evaluated. The results showed that SSPH-MPN and SSPH-PM had similar levels of AST, ALT, CRE, and BUN, all of which were lower than those in the saline-treated model group (Fig. 6C-F). These findings indicate that the optimized SSPH-MPN does not affect hepatic or renal function. Furthermore, SSPH-MPN did not cause pathological damage to major organs compared to the model group, as evidenced by the H&E images (Fig. 6G).

## Conclusion

In this study, we employed a machine learning-aided hybrid method to screen bioactive compounds from *Salvia miltiorrhiza* and *Carthamus tinctorius* for AS treatment. This multi-faceted strategy integrated network pharmacology analysis, QSAR modeling enhanced by machine learning, and molecular docking simulations, which collectively identified SAA, SAB, PCA, and HSYA as core components. To enhance their anti-AS efficacy, we developed an MPN-based codelivery system (SSPH-MPN) tailored to the structural properties of these polyphenols. Given their antioxidative potential, the dose-effect relationship of SSPH-MPN was systematically optimized based on the median-effect principle, with the intracellular ROS scavenging rate as the efficacy metric. This optimization yielded an ideal synergistic ratio (SAA: SAB: PCA: HSYA = 1:2:1:1) based on their respective D_m_ values. To validate the successful formation of the optimized SSPH-MPN, comprehensive characterization was conducted through molecular dynamics simulations, UV-vis spectroscopy, DLS, TEM, FTIR, XPS, and ICP-MS. Organ distribution studies revealed preferential accumulation in atherosclerotic lesions, supporting its targeted therapeutic action.

To evaluate the therapeutic potential of the optimized SSPH-MPN against AS, a comprehensive assessment was conducted across in vitro and in vivo models. Owing to the pivotal role of oxidative stress in early AS, the antioxidative capacity of SSPH-MPN was initially systematically evaluated. In vitro assays, including DPPH and superoxide anion scavenging tests, confirmed dose-dependent ROS suppression. Enhanced activities of antioxidant enzymes (SOD: 101.4%, GSH-Px: 12.3%), reduced lipid peroxidation (MDA: 27.2%), and decreased in situ ROS levels in apoE^-/-^ mice further corroborated its antioxidative efficacy. Besides, it was found that the optimized quaternary SSPH-MPN was capable of modulating lipid metabolism by inhibiting DiI-oxLDL uptake, mediating cholesterol efflux, reducing serum LDL-C (66.8%) and TC (35.9%) levels, and enhancing HDL-C (130.4%) level. The anti-inflammatory activity of the optimized quaternary SSPH-MPN was further supported by significant decreases in pro-inflammatory cytokine expression (IL-1β: 26.7%, IL-6: 20.4%, TNF-α: 26.9%). The potent combined anti-oxidative and anti-inflammatory effect of the optimized quaternary SSPH-MPN leads to reduced plaque burden (30.0% vs. model group) as evidenced by Oil Red O staining. Furthermore, biocompatibility was assessed through hemolysis, cytotoxicity assay, and in vivo safety studies, confirming its suitability as a safe therapeutic agent.

In conclusion, this work not only identified four synergistic anti-AS polyphenols from traditional herbs but also established an MPN-based codelivery system, providing a robust strategy for combating AS through oxidative stress mitigation, inflammation suppression, and lipid homeostasis restoration. Meanwhile, this work establishes a comprehensive paradigm from drug discovery to formulation development, combining machine learning-driven compound screening with nanotechnology-enabled codelivery for synergistic anti-AS therapy.

## Supplementary Information

Below is the link to the electronic supplementary material.

Supplementary Material 1

## Data Availability

Data and materials supporting the findings of this study are available from the corresponding author on reasonable request.

## Abbreviations

AS: Atherosclerosis
TCM: Traditional Chinese medicine
QSAR: Quantitative activity relationship
PPI: Protein-protein interaction
MPN: Metal-phenolic network
SAA: Salvianic acid A
SAB: Salvianolic acid B
PCA: Protocatechuic aldehyde
HSYA: Hydroxysafflor yellow A
SSPH-PM: Physical mixture of SAA, SAB, PCA, and HSYA
SSPH-MPN: SAA, SAB, PCA, and HSYA-loaded metal-phenolic network
UV-vis: Ultraviolet-visible spectrophotometry
FTIR: Fourier transform infrared spectroscopy
XPS: X-ray photoelectron spectroscopy
TEM: Transmission electron microscope
DLS: Dynamic light scattering
ICP-MS: Inductively coupled plasma mass spectrometry
MTT: 3-(4, 5-Dimethylthiazol-2-yl)-2,5-diphenyltetrazolium bromide
ICP-MS: Inductively coupled plasma mass spectrometry
R123: Rhodamine 123
EE: Drug entrapment efficiency
DL: Drug loading efficiency
NADH: Nicotinamide adenine dinucleotide
PMS: Phenazine methosulphate
NBT: Nitroblue tetrazolium
CLSM: Confocal laser scanning microscopy
D_m_: Median-effect dose
DHE: Dihydroethidium
MDA: Malondialdehyde
SOD: Super oxide dismutase
GSH-Px: Glutathione peroxidase
T-AOC: Total antioxidant capacity
TC: Total cholesterol
TG: Triglyceride
LDL-C: Low density lipoprotein cholesterol
HDL-C: High density lipoprotein cholesterol

## Data availability

The datasets generated during and/or analysed during the current study are available from the corresponding author on reasonable request.
