# Supplementary material for: Precision engineering of anti-atherosclerotic herbal nanomedicine: from machine learning-aided active components screening to optimized metal-phenolic network codelivery
Source: Drug Deliv Transl Res. 2025 Dec 4;16(8):2788–806. doi: 10.1007/s13346-025-02023-3 (PMC13346160; doi:10.1007/s13346-025-02023-3)
Supplement: Supplementary file 1 — Supplementary Material 1 [file 13346_2025_2023_MOESM1_ESM.docx]

Supporting Information for

Precision Engineering of Anti-Atherosclerotic Herbal Nanomedicine: From Machine Learning-Aided Active Components Screening to Optimized Metal-Phenolic Network Codelivery

Yao Chen^1a^, Meiting Lu^1a^, Lu Zhang^a^, Errong Chang^a^, Qinglan Zhu^a^, Qianlan Xu^c^, Ziting Gao^a^, Dongmei Pan^a^, Chunyan Shen^a^, Qiang Liu^a^, Zhong Zuo^*b^, Cuiping Jiang^*a,b^

*^a^ Guangdong Provincial Key Laboratory of Chinese Medicine Pharmaceutics, School of Traditional Chinese Medicine, Southern Medical University, Guangzhou 510515, People’s Republic of China.*

*^b^ School of Pharmacy, Faculty of Medicine, The Chinese University of Hong Kong, Hong Kong SAR, People’s Republic of China*

*^c^Laboratory Animal Center, Southern Medical University, Guangzhou 510515, China.*

*^1^Contributed equally to this work*

**Corresponding authors*

### *Email addresses: cuipingjiangcpu[@1](mailto:@i.smu.edu.cn)63.com (C.P., J), [joanzuo@cuhk.edu.hk](mailto:joanzuo@cuhk.edu.hk) (Z, Z)*

### **Table S1.** Modules of the protein-protein interaction network of common targets of *Salvia miltiorrhiza* and *Carthamus tinctorius* with atherosclerosis.

### **Table S2.** Description of the different machine learning methods.

### **Table S3.** Search results of active small molecule samples for each target in the chEMBL database.

### **Table S4.** Results for LsBoost machine learning models on the training set and the validation set.

### **Table S5.** Target gene protein and related PDB DOI.

### **Table S6.** Prediction of active ingredients and molecular docking results with score rankings.

### **Table S7.** Quantitative fluorescence analysis of individual organs based on area of interest (AOI) selection (n=3).

**Figure S1.** t-SNE visualization of descriptors in QSAR models.

**Figure S2.** Stage 1 hyperparameter optimization performance heatmaps of R² values across six MinLeafSize settings.

**Figure S3.** 3D scatter plot of Stage 1 results across three hyperparameters, colored by R² .

**Figure S4.** Performance comparison of the LsBoost model using default, Stage 1, and Stage 2 optimal parameters.

**Figure S5.** 2D scatter plot of Stage 2 results across three hyperparameters, colored by R².

**Figure S6.** Top ten descriptors ranked by importance in each QSAR model.

### **Figure S7.** Cellular uptake and internalization of R123-SSPH-MPN in RAW264.7 Cells.

### **Figure S8.** *In vitro* antioxidative activity results.

### **Table S1.** Modules of the protein-protein interaction network of common targets of *Salvia miltiorrhiza* and *Carthamus tinctorius* with atherosclerosis.

| Modlue | subcategory | Description | Ratio | q | MCODE | Node | Edge |
| --- | --- | --- | --- | --- | --- | --- | --- |
| 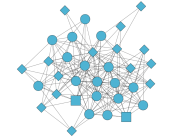 | Cardiovascular disease | Lipid and atherosclerosis | 35/127 | 6.01E-26 | 11.8 | 35 | 194 |
| 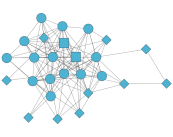 | Endocrine and metabolic disease | AGE-RAGE signaling pathway in diabetic complications | 27/127 | 6.01E-26 | 9.6 | 26 | 120 |
| 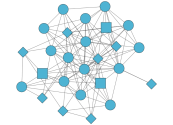 | Cardiovascular disease | Fluid shear stress and atherosclerosis | 29/127 | 1.44E-24 | 9.2 | 27 | 116 |

### **Table S2.** Description of the different machine learning methods.

| **Model** | **Abbreviation** | **description** | **parameter** |
| --- | --- | --- | --- |
| Least-Squares Boosting | LsBoost | LsBoost is a boosting algorithm for regression that minimizes squared residuals. It iteratively adds weak models, like decision trees, to correct previous errors. LsBoost helps reduce overfitting, making it ideal for continuous predictions in fields like financial modeling, risk management, and machine learning. | NumCycles =200, LearnRate =0.50, MinLeafSize  =10 |
| Gaussian Process Regression | GPR | GPR is a non-parametric, probabilistic model widely used in machine learning for regression tasks. It represents data as a collection of random variables with a joint Gaussian distribution, enabling the model to generate a distribution over potential functions that can explain the observed data. | Noise standard deviation (σ) ,0.1 |
| Support Vector Machine | SVM | SVM is a machine learning algorithm that uses supervised learning to solve classification, regression, and outlier detection problems. SVMs are known for their ability to find the optimal decision boundary between classes by maximizing the margin between them. | Default |
| Decision Tree | DT | DT model is a classical ML based model which categorizes the data into various subsets to identify the potential structure, patterns and relationships among the data. | leaf node,10 |
| Random Forest | RF | RF is an ensemble learning method for classification, regression and other tasks that works by creating a multitude of decision trees during training. For classification tasks, the output of the random forest is the class selected by most trees. For regression tasks, the output is the average of the predictions of the trees.Random forests correct for decision trees' habit of overfitting to their training set. | Tree depth,500;  leaf node,5 |
| Partial Least Squares | PLS | PLS model is a regression technique that decomposes data into latent variables to explain the covariance between a set of experimental variables and neural responses. | Default |

### **Table S3.** Search results of active small molecule samples for each target in the chEMBL database.

| **Target gene** | **Target name** | **Activity type** | **Number** |
| --- | --- | --- | --- |
| APOB | Apolipoprotein B | IC50 | 49 |
| ACE | Angiotensin I Converting Enzyme | IC50 | 12 |
| AKT1 | RAC-alpha serine/threonine-protein kinase | IC50 | 37 |
| BCL2 | Apoptosis regulator Bcl-2 | IC50 | 30 |
| BCL2L1 | Bcl2-antagonist of cell death | IC50 | 1 |
| CASP3 | Caspase-3 | IC50 | 700 |
| CASP8 | Caspase-8 | IC50 | 14 |
| CASP9 | Caspase-9 | IC50 | 10 |
| CCL2 | C-C Motif Chemokine Ligand 2 | IC50 | 23 |
| CD40LG | CD40 ligand | IC50 | 1 |
| CXCL2 | C-X-C motif chemokine 2 | Kd | 3 |
| CHUK | Inhibitor of nuclear factor kappa-B kinase subunit alpha | IC50 | 13 |
| CYP1A1 | Aromatase | inhibition | 82 |
| FOS | Protein c-Fos | inhibition | 2 |
| GSK3B | Glycogen synthase kinase-3 beta | inhibition | 2 |
| ICAM1 | Intercellular adhesion molecule 1 | IC50 | 156 |
| IKBKB | Inhibitor of nuclear factor kappa-B kinase subunit beta | inhibition | 1090 |
| IKKA | Inhibitor of nuclear factor kappa-B kinase subunit alpha | IC50 | 13 |
| IL6 | Interleukin-6 | IC50 | 17 |
| MAPK1 | Mitogen-activated protein kinase 1 | inhibition | 5 |
| MAPK14 | Mitogen-activated protein kinase 14 | inhibition | 2 |
| MAPK8 | Mitogen-activated protein kinase 8 | inhibition | 2 |
| MMP1 | Interstitial collagenase | Ratio IC50 | 70 |
| NCF2 | NaN | NaN | NaN |
| NFE2L2 | Nuclear factor erythroid 2-related factor 2 | Activity | 7 |
| NFKBIA | NF-kappa-B inhibitor alpha | IC50 | 12 |
| NOS3 | Nitric oxide synthase 3 | IC50 | 1 |
| PPARG | Peroxisome proliferator-activated receptor gamma | IC50 | 14 |
| RELA | Transcription factor p65 | IC50 | 28 |
| RXRA | NaN | NaN | NaN |
| SELE | E-selectin | IC50 | 113 |
| STAT3 | Signal transducer and activator of transcription 3 | IC50 | 14 |
| TNF | Tumor necrosis factor | IC50 | 349 |
| TP53 | Cellular tumor antigen p53 | inhibition | 11 |
| VCAM1 | Vascular cell adhesion protein 1 | IC50 | 93 |

### **Table S4.** Results for LsBoost machine learning models on the training set and the validation set.

| **Target** | **MSE** | | **MAE** | | **RMSE** | | **R2** | |
| --- | --- | --- | --- | --- | --- | --- | --- | --- |
|  | Training Set | Validation Set | Training Set | Validation Set | Training Set | Validation Set | Training Set | Validation Set |
| AKT1 | 0.011 | 0.000 | 0.049 | 0.009 | 0.106 | 0.012 | 0.987 | 1.000 |
| APOB | 0.076 | 0.078 | 0.162 | 0.155 | 0.275 | 0.279 | 0.816 | 0.824 |
| CASP3 | 0.136 | 0.084 | 0.269 | 0.221 | 0.369 | 0.290 | 0.890 | 0.934 |
| CCL2 | 0.001 | 0.000 | 0.024 | 0.014 | 0.035 | 0.017 | 0.994 | 0.999 |
| ICAM1 | 0.041 | 0.001 | 0.075 | 0.017 | 0.202 | 0.022 | 0.930 | 0.999 |
| IKBKB | 0.089 | 0.060 | 0.188 | 0.162 | 0.298 | 0.244 | 0.869 | 0.915 |
| IL6 | 0.004 | 0.001 | 0.043 | 0.026 | 0.062 | 0.033 | 0.996 | 0.999 |
| MMP1 | 0.011 | 0.002 | 0.057 | 0.025 | 0.107 | 0.041 | 0.991 | 0.999 |
| RELA | 0.001 | 0.000 | 0.014 | 0.006 | 0.026 | 0.007 | 0.998 | 1.000 |
| SELE | 0.060 | 0.000 | 0.096 | 0.011 | 0.244 | 0.013 | 0.911 | 1.000 |
| TNF | 0.048 | 0.019 | 0.128 | 0.082 | 0.219 | 0.139 | 0.912 | 0.966 |
| VCAM1 | 0.021 | 0.000 | 0.050 | 0.006 | 0.145 | 0.009 | 0.950 | 1.000 |

### **Table S5.** Target gene protein and related PDB DOI.

| **Target gene** | **PDB ID** | **PDB DOI** |
| --- | --- | --- |
| AKT1 | 2UZT | https://doi.org/10.2210/pdb2UZT/pdb |
| CASP3 | IRE1 | https://doi.org/10.2210/pdb1RE1/pdb |
| CCL2 | 4USO | https://doi.org/10.2210/pdb4USO/pdb |
| ICAM1 | 6S8U | https://doi.org/10.2210/pdb6S8U/pdb |
| IKBKB | 4KIK | https://doi.org/10.2210/pdb4KIK/pdb |
| RELA | 5URN | https://doi.org/10.2210/pdb5URN/pdb |
| SELE | 8R5L | https://doi.org/10.2210/pdb8R5L/pdb |
| MMP1 | 1SU3 | https://doi.org/10.2210/pdb1SU3/pdb |
| TNF | 6X81 | https://doi.org/10.2210/pdb6X81/pdb |
| VCAM1 | 1VSC | https://doi.org/10.2210/pdb1VSC/pdb |

### **Table S6.** Prediction of active ingredients and molecular docking results with score rankings.

| **Name** | **Score** | **Score1** | **Socre2** | **Predicted Activity Rank** | **Binding Affinity Rank** | **Score Rank** |
| --- | --- | --- | --- | --- | --- | --- |
| lignan | 0.98 | 8.42 | -8.75 | 37 | 1 | 1 |
| Salvianolic acid B | 0.95 | 8.60 | -7.91 | 4 | 2 | 2 |
| Salvianolic acid E | 0.94 | 8.51 | -7.87 | 8 | 3 | 3 |
| Dihydroisotanshinone I | 0.91 | 8.47 | -7.40 | 18 | 4 | 4 |
| Hydroxysafflor yellow A | 0.91 | 8.61 | -7.15 | 2 | 12 | 5 |
| Salvianolic acid H | 0.91 | 8.45 | -7.35 | 25 | 6 | 6 |
| Hydroxysafflor yellow B | 0.90 | 8.39 | -7.37 | 48 | 5 | 7 |
| Salvianolic acid C | 0.90 | 8.68 | -7.05 | 1 | 16 | 8 |
| Rutin | 0.90 | 8.49 | -7.24 | 11 | 7 | 9 |
| Apigenin 6,8-di-C-β-D-glucopyranoside | 0.90 | 8.52 | -7.19 | 7 | 11 | 10 |
| tagetiin | 0.90 | 8.46 | -7.23 | 20 | 8 | 11 |
| Acacetin 7-O-β-D-glucuronide | 0.90 | 8.46 | -7.20 | 24 | 9 | 12 |
| Saffloquinoside C | 0.90 | 8.59 | -7.04 | 5 | 17 | 13 |
| ursolic acid | 0.90 | 8.39 | -7.20 | 47 | 10 | 14 |
| Kaempferol 3-rutinoside | 0.89 | 8.48 | -7.07 | 12 | 15 | 15 |
| 4,8-Dimethylphenanthro | 0.89 | 8.45 | -7.08 | 29 | 13 | 16 |
| Isotanshinone II | 0.89 | 8.43 | -7.07 | 34 | 14 | 17 |
| Cynaroside | 0.88 | 8.61 | -6.78 | 3 | 20 | 18 |
| Danshenol B | 0.88 | 8.46 | -6.87 | 22 | 18 | 19 |
| Rhodiosin | 0.88 | 8.46 | -6.84 | 19 | 19 | 20 |
| Quercitrin | 0.87 | 8.57 | -6.66 | 6 | 28 | 21 |
| Hyperoside | 0.87 | 8.43 | -6.77 | 32 | 21 | 22 |
| EIC | 0.87 | 8.41 | -6.77 | 38 | 22 | 23 |
| 6-Hydroxykaempferol 3-O-β-D-glucoside | 0.87 | 8.45 | -6.73 | 27 | 23 | 24 |
| Salvianolic acid A | 0.87 | 8.46 | -6.69 | 23 | 26 | 25 |
| Oleanolic acid deriv. | 0.87 | 8.44 | -6.69 | 31 | 25 | 26 |
| Carthamin | 0.87 | 8.43 | -6.69 | 35 | 24 | 27 |
| Astragalin | 0.87 | 8.48 | -6.62 | 13 | 30 | 28 |
| Salvianolic acid D | 0.87 | 8.43 | -6.67 | 36 | 27 | 29 |
| Salvianolic acid I | 0.87 | 8.45 | -6.65 | 26 | 29 | 30 |
| Tanshinone I | 0.85 | 8.40 | -6.45 | 43 | 31 | 31 |
| (+)-Syringaresinol | 0.85 | 8.40 | -6.45 | 44 | 32 | 32 |
| isotanshinone iib | 0.82 | 8.41 | -5.85 | 40 | 34 | 33 |
| Salvianolic acid G | 0.82 | 5.85 | -5.85 | 51 | 33 | 34 |
| Monomethyl lithospermate | 0.81 | 8.46 | -5.70 | 21 | 35 | 35 |
| Danshenol | 0.81 | 8.47 | -5.66 | 15 | 36 | 36 |
| Spirostan-3-ol, (3beta,5alpha,25S)- | 0.81 | 8.41 | -5.65 | 41 | 37 | 37 |
| Hydroxysafflor yellow C | 0.81 | 8.39 | -5.65 | 49 | 38 | 38 |
| carnosol | 0.80 | 8.47 | -5.50 | 16 | 39 | 39 |
| przewalskin b | 0.80 | 8.47 | -5.48 | 14 | 40 | 40 |
| isosalvianolic acid c | 0.80 | 8.41 | -5.48 | 39 | 41 | 41 |
| lirioresinol-A | 0.79 | 8.40 | -5.41 | 45 | 42 | 42 |
| oleanolic acid | 0.79 | 8.39 | -5.40 | 46 | 43 | 43 |
| Lithospermic acid | 0.79 | 8.44 | -5.32 | 30 | 44 | 44 |
| dihydrotanshinone | 0.79 | 8.50 | -5.23 | 9 | 46 | 45 |
| Methylenetanshinquinone | 0.79 | 8.47 | -5.26 | 17 | 45 | 46 |
| Dihydrotanshinone i | 0.79 | 8.50 | -5.21 | 10 | 47 | 47 |
| Isoquercitrin | 0.78 | 8.43 | -5.20 | 33 | 48 | 48 |
| tigogenin | 0.78 | 8.41 | -5.17 | 42 | 49 | 49 |
| 6-hydroxykaempferol-3-O-beta-D-glucoside | 0.78 | 8.45 | -5.13 | 28 | 50 | 50 |

### **Table S7.** Quantitative fluorescence analysis of individual organs based on area of interest (AOI) selection (n=3).

| **Organ** | **Sum Intensity** | | | | | |
| --- | --- | --- | --- | --- | --- | --- |
|  | **Free DiR** | | | **DiR-SSPH-MPN** | | |
| **Aorta** | 103010 | 125670 | 117814 | 2809530 | 2678145 | 2768910 |
| **Heart** | 233399 | 197690 | 209159 | 1574562 | 1908426 | 898247 |
| **Lung** | 251615 | 308225 | 309152 | 3881416 | 4195801 | 3187192 |
| **Spleen** | 748974 | 636118 | 636848 | 3311059 | 4354109 | 1316002 |
| **Kidney** | 419201 | 463903 | 484676 | 3952068 | 4102728 | 2482769 |
| **Liver** | 1271559 | 1410617 | 1537806 | 15245042 | 15058700 | 19258136 |
| **Totoal Intensity** | 3027758 | 3142223 | 3295455 | 30773677 | 32297909 | 29911256 |
| **Plaque Targeting rate (%)** | 3.40 | 4.00 | 3.58 | 9.13 | 8.29 | 9.26 |





**Figure S1.** t-SNE visualization of descriptors in QSAR models. For each QSAR model, the left panel shows t-SNE projections of the training set (blue) and test set (red), while the right panel displays t-SNE visualization colored by R^2^ values.


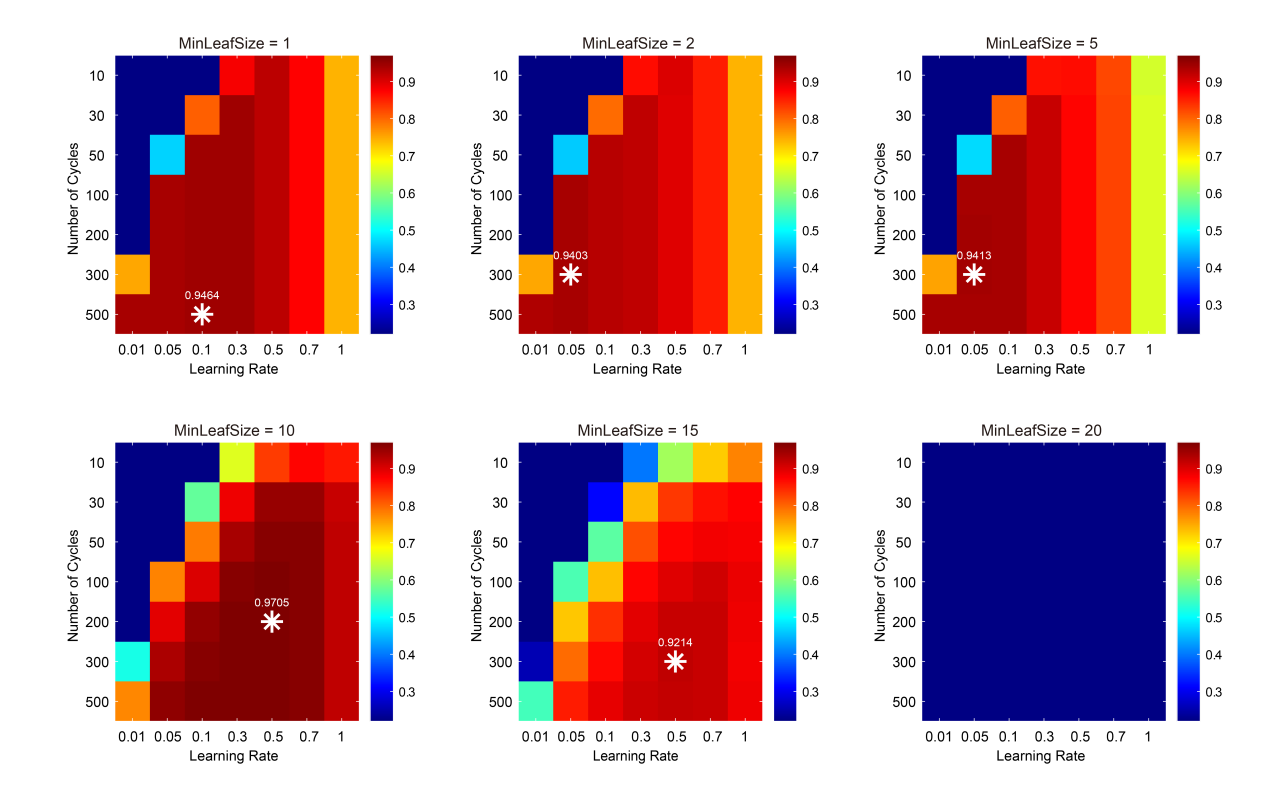


**Figure S2.** Stage 1 hyperparameter optimization performance heatmaps of R² values across six MinLeafSize settings (1,2,5,10,15 and 20), with learning rate and number of cycles. Color scale: blue (low) to red (high performance refers to R² value). White stars mark optimal configurations.


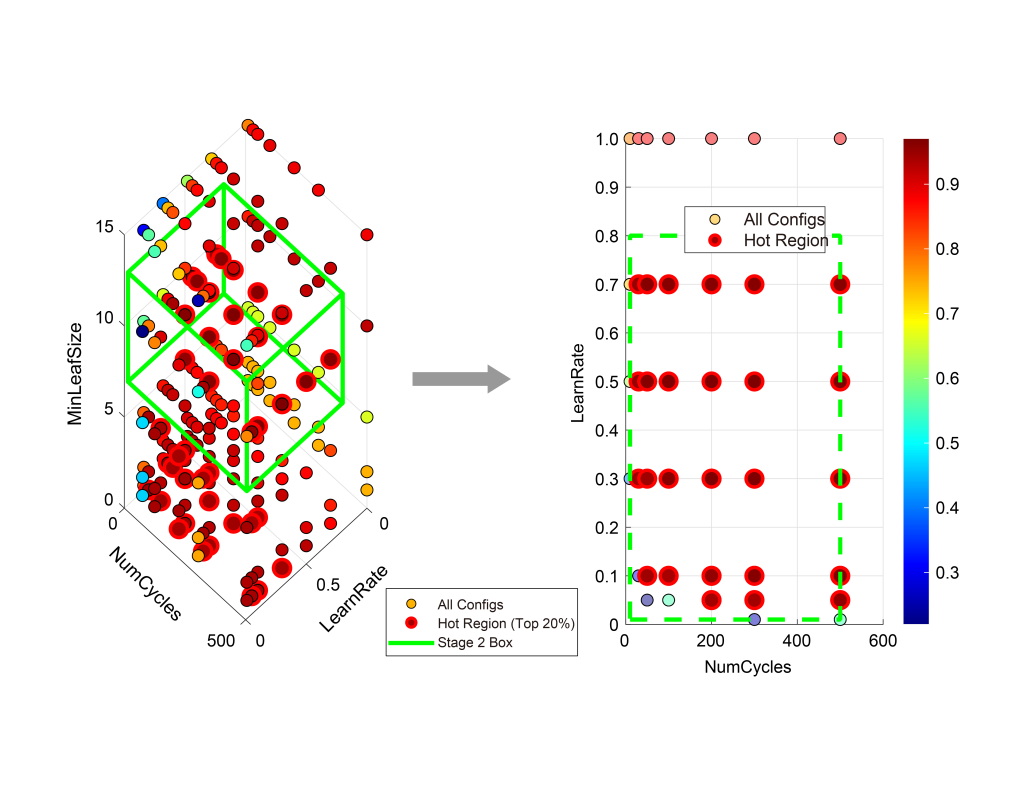


**Figure S3.** 3D scatter plot of Stage 1 results across three hyperparameters, colored by R² (blue: low, red: high). Red points indicate top performers; green box marks Stage 2 search space. 2D projection on NumCycles-LearnRate plane with Stage 2 boundaries (green dashed box).


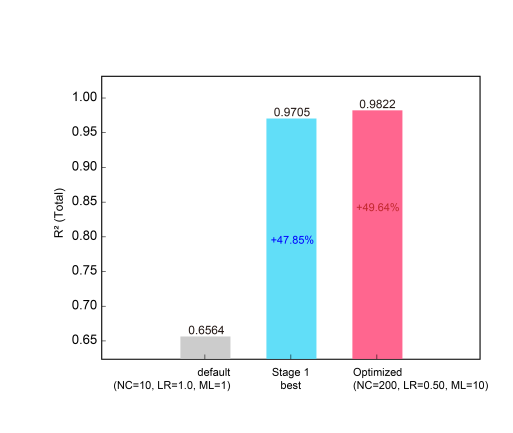


**Figure S4.** Performance comparison of the LsBoost model using default, Stage 1, and Stage 2 optimal parameters.


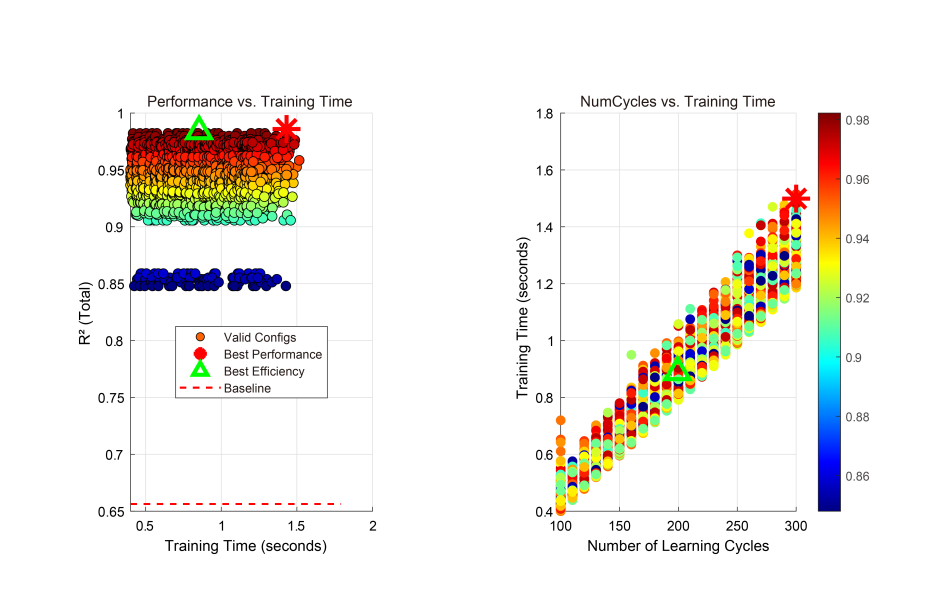


**Figure S5.** 2D scatter plot of Stage 2 results across three hyperparameters, colored by R². Red points marks the best performance parameter configuration; green box marks the optimized parameter configuration.


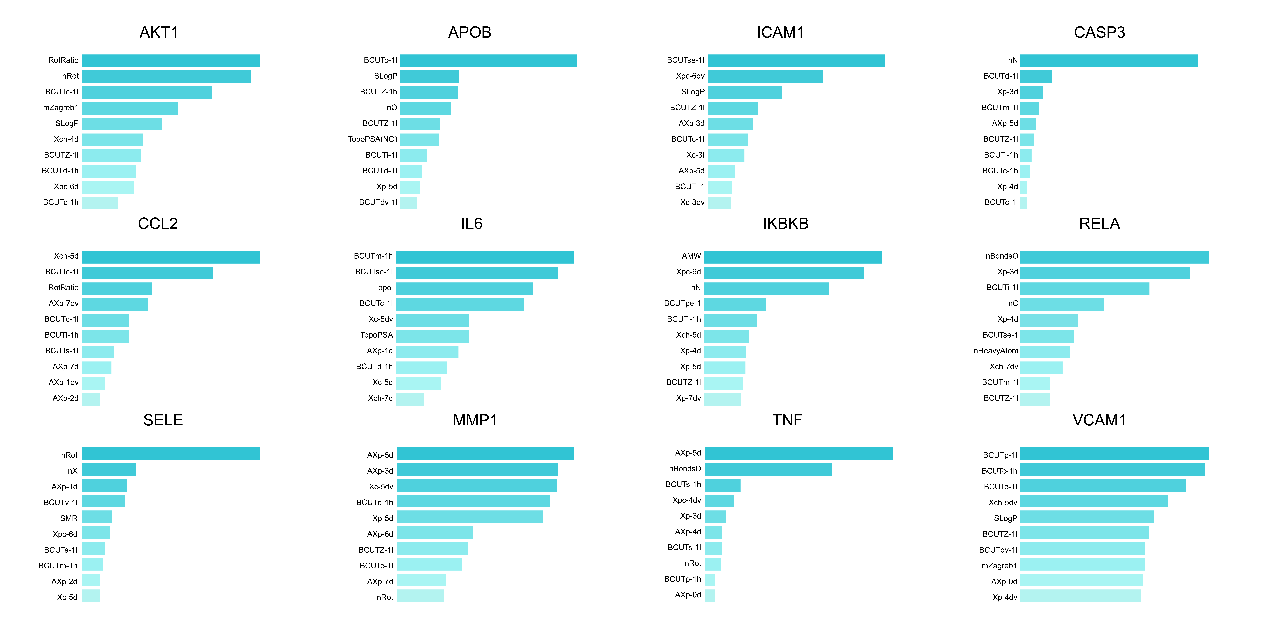


**Figure S6.** Top ten descriptors ranked by importance in each QSAR model.


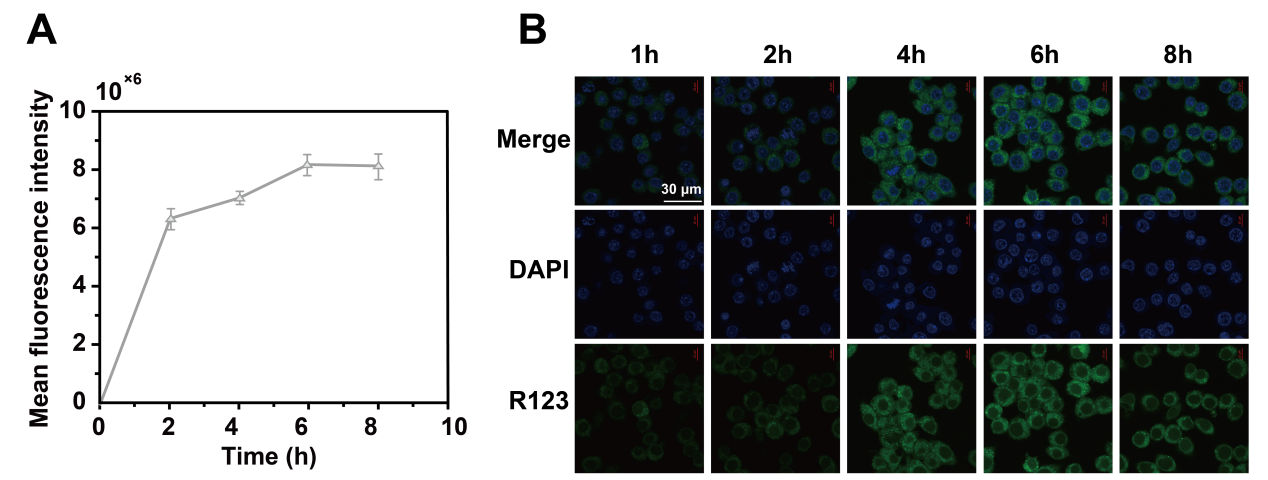


**Figure S7.** (A) Flow cytometry analysis of internalized R123-SSPH-MPN formulated with 1:2:1:1 upon D_m_ values at the predetermined time points (n = 6). (B) Confocal images of RAW264.7 cells incubated with the R123-SSPH-MPN formulated with 1:2:1:1 upon D_m_ values for various lengths of time. Green fluorescence represents the R123-SSPH-MPN. Blue fluorescence is nucleus staining with DAPI. Scale bar: 30 μm.


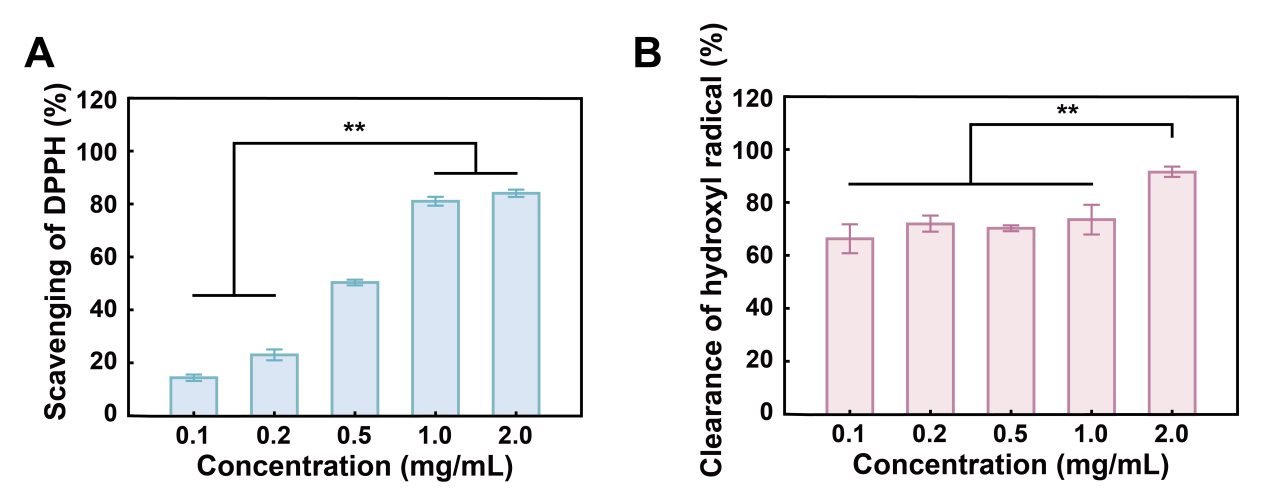


**Figure S8.** (A) Scavenging of DPPH for SSPH-MPN at the concentrations of 0.1, 0.2, 0.5, 1.0 to 2.0 mg/mL (n=6, ***p<0.01)*. (B) Scavenging of hydroxyl radical for SSPH-MPN at the concentrations of 0.1, 0.2, 0.5, 1.0 to 2.0 mg/mL (n=6, ***p<0.01)*.
